# Effects of a virtual iSupport Program on carers and people with dementia

**DOI:** 10.1002/alz.70747

**Published:** 2025-09-29

**Authors:** Lily Xiao, Shahid Ullah, Ying Yu, Claudia Meyer, Michael Chapman, Langduo Chen, Kai Ping TAN, Sue McKechnie, Mel Ottaway, Andre Queiroz De Andrade, Julie Ratcliffe, Craig Whitehead, Kam Tran, Yao Wang, Alison Kitson

**Affiliations:** ^1^ College of Nursing and Health Sciences Flinders University Adelaide South Australia Australia; ^2^ College of Medicine and Public Health Flinders University Adelaide South Australia Australia; ^3^ Bolton Clarke Research Institute Victoria Australia; ^4^ Canberra Health Services Garran ACT Australia; ^5^ Southern Adelaide Local Health Network Flinders Dr Adelaide South Australia Australia; ^6^ Community Services Resthaven Incorporated Adelaide South Australia Australia; ^7^ Quality Use of Medicines and Pharmacy Research Centre University of South Australia Adelaide South Australia Australia; ^8^ Xiangya School of Nursing Central South University Changsha Hunan China; ^9^ International Learning Collaborative Adelaide South Australia Australia

**Keywords:** behavioral problem, dementia, family caregivers, health services for the aged, home care services, online social support, quality of life, self‐efficacy

## Abstract

**INTRODUCTION:**

We conducted a virtual iSupport Program intervention for carers of people living with dementia (PLWD).

**METHODS:**

We applied a pragmatic randomized controlled trial to evaluate a multicomponent program delivered virtually in four organizations (July 2022 to December 2024). The primary outcome was quality of life (QoL) of carers and PLWD at 12 months post‐baseline, and the secondary outcomes were carers’ self‐efficacy, social support, reactions to behavior, PLWD's behavior frequency, hospital admissions, and emergency department presentations.

**RESULTS:**

One hundred forty‐nine carers enrolled in the study. The intervention group reported increased mental‐health‐related QoL points of 12.0 (*p* < 0.001), self‐efficacy points of 14.8 to 18.5 (*p* < 0.001), social support points of 0.25 (*p* < 0.028), reduced reactions to behavior points of ‐0.25 (*p* < 0.028), and a 60% lower hospital admission rate (*p* = 0.045) at 12 months compared with the usual care group.

**DISCUSSION:**

The virtual iSupport Program showed benefits for both carers and PLWD in a 12‐month intervention.

**TRIAL REGISTRATION:**

Australia New Zealand Clinical Trials Registry: ACTRN12622000199718.

**Highlights:**

- A total of 149 dementia carers participated in the virtual iSupport program intervention trial.
- The program included skills training, peer support, and access to care services.
- The program improved mental health‐related quality of life for carers.
- The program improved self‐efficacy, social support, and reduced distress for carers.
- The program reduced 60% hospital admission rate for people with dementia.

## 1 INTRODUCTION

The number of people living with dementia (PLWD) in Australia has reached 411,100 and will more than double to 849,300 by 2058.^1^ Of those, 67% live at home and are cared for by family carers.^2^ Although family carers are recognized as crucial care partners in enabling PLWD to live at home,^2^, ^3^ they received less dementia care education and support than health and aged care professionals.^3^, ^4^ Systematic review and meta‐analyses consistently show that multicomponent intervention programs comprising dementia care training and other needs‐based support for carers are more effective for improving the health and well‐being of PLWD and carers than single component interventions.^5^, ^6^, ^7^, ^8^ Yet, the effects of virtual multicomponent interventions in pragmatic care settings remain largely unclear. The “Partnership in iSupport Program” (hereafter: iSupport program) in Australia is a virtually delivered multicomponent intervention incorporating trained facilitator‐enabled dementia care education, peer support, and needs‐based individual support for carers. The study includes three phases: (1): co‐designing the program with stakeholders^9^, ^10^; (2) evaluating the effectiveness, cost‐effectiveness, and factors affecting the implementation of the program in real care settings using a nested qualitative study alongside the randomized controlled trial (RCT); and (3) adopting the program in participating organizations. This paper reports the effectiveness of the program.

**FIGURE 1 alz70747-fig-0001:**
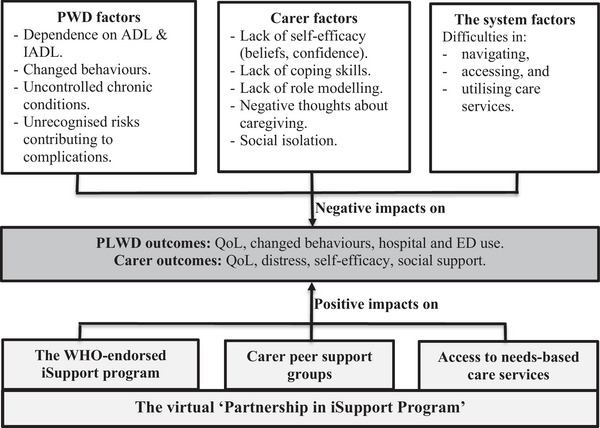
A conceptual model of change. ADL, acitivites of daily living; ED, emergency department; iADL, instrumental activities of daily living; PLWD, people living with dementia; PWD, people with dementia; QoL, quality of life.

Based on the stress and process model, factors that have negative impacts on the health outcomes for PLWD and their carers are derived from PLWD, carers, and the care system.^11^, ^12^ We outlined these three‐group factors in Figure 1, illustrated our intervention components to target these factors in a conceptual model of change and discussed details in the following sections.

Psychoeducation is a main category of carer intervention and has demonstrated improved self‐efficacy and reduced carer distress to the changed behaviors of PLWD.^5^, ^13^ The World Health Organization (WHO) iSupport for Dementia program is a comprehensive psychoeducation program^14^; and was culturally adapted in Australia prior to this trial.^15^ The Australia iSupport program includes a new module “Consumer‐directed aged care and dementia care” to enable carers to navigate and choose care services.^15^ Supplementary file 1 outlines the Australian iSupport program. However, psychoeducation has limitations in improving QoL for carers.^7^, ^13^

Peer support is another widely used intervention. Studies revealed that peer support created a safe space for carers to debrief their experiences and gain emotional support from peers; thus, they experienced emotional well‐being while reducing negative thoughts about caregiving.^16^, ^17^ Peer support groups also provided opportunities for role modelling and coaching activities which enhanced carers’ self‐efficacy in dementia care.^18^, ^19^ Two meta‐analyses confirm that facilitator‐enabled peer support groups can improve QoL for carers and their perception of social support.^20^, ^21^

Timely access to health and social care services enables PLWD to maintain health and prevent avoidable care crises, hospital admission, and emergency department (ED) use.^22^, ^23^ However, structural barriers existed in the care systems which prevented carers from accessing and using the care services.^9^, ^24^ Systematic reviews confirm that interventions comprising a trained facilitator‐enabled access to and use of care services (or care coordination) can improve the QoL, reduce changed behaviors and delay nursing home admission of PLWD.^6^, ^25^ Carers new to their role benefited most from trained facilitators who supported them at the time of dementia diagnosis^9^ or throughout the first two years in their carers’ role.^26^

**RESEARCH IN CONTEXT**

1. **Systematic review**: We undertook two systematic reviews (PROSPERO CRD42021257562, CRD42021262174) and identified that virtual psychoeducation interventions can improve carers’ mental health, reduce their stress, and empower them to perform self‐care and high‐quality dementia care, especially when the intervention was led by facilitators and had a peer support component. However, the facilitator‐enabled virtual multicomponent intervention was not evaluated in real hospital and community aged care settings.
2. **Interpretation**: The virtual “Partnership in iSupport Program” which incorporated facilitator‐enabled psychoeducation program, carer peer support, and access to needs‐based care services demonstrated improved mental‐health‐related quality of life, self‐efficacy, social support, reduced reactions to behavior for carers and a 60% lower hospital admission rate for people with dementia at 12 months compared with the usual care group.
3. **Future directions**: Implementation studies are imperative to embed the evidence‐based virtual “Partnership in iSupport Program” in routine care services and integrate the facilitator's role in health and aged care organisations.

In this study, we tested two primary hypotheses that we stated our primary hypotheses as carers in the virtual “Partnership in iSupport Program” will report improved QoL of carers and PLWD compared to those allocated to the usual care group at 12 months. We also tested these secondary hypotheses that carers in the virtual iSupport Program will report improved self‐efficacy, social support, and reactions to behaviors of PLWD; and will report reduced changed behaviors, hospital admissions, and ED presentations compared to the usual care group. We also tested the primary and secondary hypotheses at 6 months.

## 2 METHODS

### 2.1 Design

**FIGURE 2:**
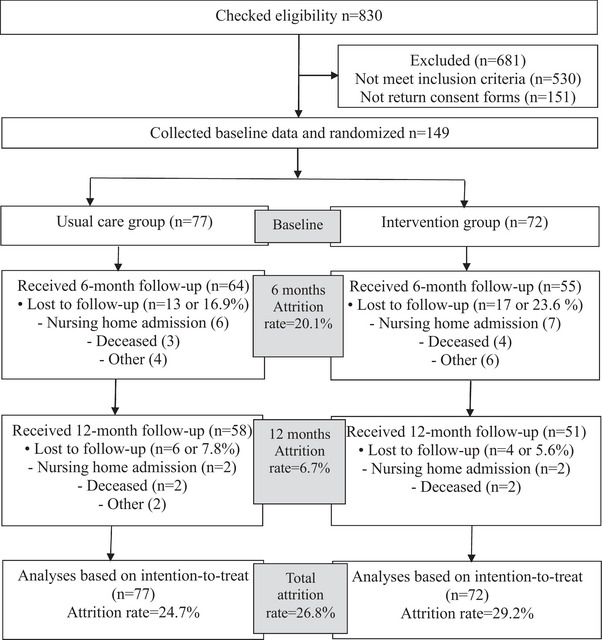
Flow chart of the virtual iSupport Program.

We undertook a two‐arm, parallel‐group, multicenter, RCT to determine the effects of the virtual iSupport Program between July 2022 and December 2024. Carer participants were recruited from two tertiary hospitals and two community aged care organizations across three states in Australia. They were assigned to either[Fig alz70747-fig-0001] the usual care group or the intervention group after baseline data collection (see Figure 2). We outlined the detailed trial processes in Supplementary file 2: CONSORT reporting checklist. This study was approved by the Southern Adelaide Clinical Human Research Ethics Committee (No. 2021/HRE00273; date: 14‐09‐2021). We detailed the study design and methods in a published study protocol^27^ and an updated study protocol in Supplementary file 3. We registered the trial on the Australia New Zealand Clinical Trials Registry: ACTRN12622000199718. We made great efforts to address diversity, equity and inclusion in the study design by engaging carers from culturally and linguistically diverse (CALD) backgrounds, respecting their diverse needs in the trial and interpreting results in the CALD population context.

### 2.2 Paticipants

In the two tertiary hospitals, we recruited carers of PLWD from memory and geriatric clinics, geriatric and rehabilitation wards. In the two aged care organizations, we recruited carers of PLWD from government‐subsidized community aged care services including the “Commonwealth Home Support Programme” which provides entry‐level care services for older people, for example, meals and food preparation, bathing and grooming, laundry, and other chores; and “Home Care Packages” which provide more complex care such as nursing care and multidisciplinary care services for older people with medical conditions and impairments, in addition to the entry‐level care services. In addition, we also advertised via social media, recruited a small proportion of carers, and assigned them to one of the participating organizations. Carers who met these inclusion criteria were invited to participate: (1) aged ≥18 years; (2) providing care at least twice a week for a care recipient living with dementia or probable dementia as assessed by a site‐specific researcher using measures for (a) cognitive impairment using the Mini‐Mental State Examination^28^ or Rowland Universal Dementia Assessment Scale^29^ or Global Deterioration Stages between Normal Ageing and Alzheimer's Disease^30^; and (b) self‐care decline and changed behaviors using the Blessed Dementia Dependence Score.^31^ Carers were excluded if they had self‐reported severe mental health conditions or terminal illness that could significantly impact their ability to participate in the study; were involved in other studies; and could not read English without additional assistance. Site‐specific researchers met the carer‐PLWD dyads face‐to‐face or online to explain the study to them and take informed consent or third‐party consent if the PLWD was unable to take consent themselves. The researchers performed the cognitive assessment for the PLWD to ascertain the carers’ eligibility to participate in the study and simultaneously collected baseline information from both carers and PLWD.

### 2.3 Randomization

Carers were randomly assigned to the usual care group or the intervention group using permuted block randomization with a fixed block size of four. To ensure balance, randomization was stratified by carer relationship (spouse vs. non‐spouse) and care recipient dementia severity (mild vs. moderate). The severity of dementia was determined using the Global Deterioration Stages between Normal Ageing and Alzheimer's Disease.^30^ The allocation sequence was generated by an independent statistician (S.U.) who had no role in recruitment, intervention delivery, or outcome assessment using R software version 4.3.2. Assignments were placed in sequentially numbered, opaque, and sealed envelopes, which were stored securely and opened only after each participant completed baseline measures, thus preserving concealment until allocation.

### 2.4 Procedures

We developed a comprehensive project implementation manual to standardize the intervention implementation of the program across multiple sites. iSupport facilitators, which included two registered nurses and two social care professionals, were internally selected by the four participating organizations and trained by the project team to deliver the intervention. We outlined “The training and support for facilitators” in Supplementary file 4. They recorded their intervention activities, monitored the process and outcomes, discussed these with site‐specific leaders (two registered nurses, one geriatrician, and one physiotherapist) in regular meetings and submitted the records to the project team as part of data for analysis. In addition, we held fortnightly meetings with the facilitators to share experiences with others and gain advice from the project team.

Carers in the intervention group were assigned to a trained facilitator to receive these interventions: (1) Psychoeducation: The facilitator encouraged carers to undertake a learning needs assessment using a validated assessment tool, then selecting 20 out of 30 units from the iSupport program in their study plan for self‐directed learning; (2) Peer support group: The facilitator organized monthly online meetings using the Zoom video conference platform based on “A meeting agenda for peer support meetings” (see Supplementary file 5), encouraging and motivating carers to share their experiences of the program with others and providing support to each other. The facilitator also arranged a mobile application WhatsApp group, sending weekly text messages as motivation for their study plan, and encouraged carers to connect with each other for support; (3) Needs‐based support: The facilitator encouraged carers to make requests for individual support by phone, particularly during transition of care settings, for support with changed behaviors or complications of PLWD, and when experiencing emotional stress. Carers in the usual care group received support provided by Dementia Australia or other publicly funded carer support services. They were reminded monthly by email or text message by the site‐specific researcher to access these care resources and services.

### 2.5 Outcome measures

Our primary outcome measures were QoL of carers and PLWD. We used the Short Form 12 health survey questionnaire (SF‐12) to measure the QoL of carers.^32^ The tool includes six items to measure physical‐health‐related QoL (SF‐12 PCS) and six items to measure mental‐health‐related QoL domains. We transformed the raw scale scores of the SF‐12 to a range of 0–100 scale for the physical‐health‐related QoL and mental‐health‐related QoL domains, respectively.^33^ A higher score indicates better QoL. We employed the Quality of Life in Alzheimer's Disease‐Proxy (QOL‐AD‐Proxy) to measure the QoL for PLWD.^34^ This scale has 13 items with a score ranging from 1 to 52. A higher score indicates better QoL.

We also included secondary outcome measures in the study as detailed in the follows. We used the Revised Scale for Caregiving Self‐Efficacy Scale (RSCSE)^35^ which includes 15 items to measure carers’ confidence of undertaking care activities in these three domains, with each domain including five items: obtaining respite, responding to changed behaviors of PLWD and controlling upsetting thoughts about caregiving. The score for each item ranges from 0 to 100. A higher score indicates better self‐efficacy. We applied the Quality of Social Support domain in the Older People in Europe Index (COPE Index‐QS) to measure carers’ perceived social support.^36^ This scale includes five items with a score range from 1 to 4 on each item. A higher score indicates better social support. We used the Revised Memory and Behavior Problem Checklist (RMBPC) to measure two domains: PLWD's changed behaviors frequency and carers’ reactions to those behaviors.^37^ This scale includes 24 items in each domain with a score ranging from 0 to 4 on each item. A higher score on the RMBPC frequency indicates more changed behaviors of PLWD. A higher score on RMBPC reaction indicates more carers’ distress reactions to the changed behaviors of PLWD. Carers undertook an online or hardcopy survey based on their preferences at three time points: baseline, 6 months, and 12 months. We measured hospital admissions and emergency department presentations for PLWD at baseline and through monthly follow‐up surveys for 12 months using the Resource Utilization in Dementia (RUD) Questionnaire.^38^ We requested carers to report iSupport unit completion at 6 months and 12 months as evidence of their adherence to the study plan. We also measured carers’ satisfaction with support provided by the trained facilitators using 10 statements about activities delivered by the facilitators as per study protocol. Each statement was rated on a 5‐Likert scale with higher scores representing higher satisfaction with the support.

### 2.6 Statistical analysis

We calculated the sample size based on an effect size (Cohen's *d*) of 0.57 and a standard deviation of 8.63 in the mental‐health‐related QoL of carers in a multicomponent intervention using RCT.^39^ We assumed 50 carers per group to achieve 80% power to detect a difference between means of 4.93 with a pooled standard deviation of 8.63 (medium effect size of 0.57) in the RCT. Considering a 40% attrition rate in a 12‐month RCT, we planned to recruit 140 carers to the trial with approximately 70 carers in each group. A Stata code *power* was used to calculate the sample size.

We entered data into SPSS 29 for descriptive data analysis. The missing values were filled using multiple imputation. Data were analyzed on an intention‐to‐treat basis based on group assignments. Two researchers (S.U. and Y.W.) who were blinded to the data collection and group assignment undertook the data analysis. Baseline demographic and clinical characteristics were compared using independent samples t tests for continuous variables and chi‐squared tests (or Fisher's exact tests) for categorical variables.

We used two‐level mixed‐effects regression models with a random intercept for each participant to account for the nested data structure (repeated measures at baseline, 6 months, and 12 months within individuals) using Stata version 18.^40^ This approach accommodates within subject correlation and increases the efficiency of treatment effect estimates at 12 months. We used the maximum likelihood estimate procedure to compare significant differences in outcomes over time and between groups. Moreover, we used a mixed‐effect Poisson regression model to determine the incidence rate ratio for hospital admission and emergency department presentation of PLWD between the intervention and the usual care groups. Findings from the multivariate mixed effect linear and the Poisson regression model were adjusted for baseline covariates including gender, age, relationship with the care recipients, marital status, duration in carer role, live in the same household with PLWD or not, religion, employment status, education level, financial pressure, family members’ help, and baseline outcome measures. We applied the two‐sided test for all analyses and set the level of significance at *α* = 0.05.

As an intervention focused on dementia training and social support for carers, the virtual iSupport Program was viewed as a low‐risk program. We did not have a data safety monitoring board. The Steering Committee formed by chief investigators monitored adverse events during the trial.

### 2.7 Changes to the trial protocol

We undertook an internal pilot study to explore the recruitment, retention, and adherence to the study protocol. Results in the pilot study informed minor changes detailed as follows. First, to address recruitment difficulties, we used social media to recruit carers of PLWD in addition to the proposed recruitment strategy from the pool of existing clients of the four participating organizations. Second, we expanded the inclusion criteria to include carers of people with probable dementia as detailed in Section 2.2. “Participants” to address that up to 63.7% of PLWD did not have a formal diagnosis.^41^ Third, we removed the inclusion criteria that required carers to have access to the internet via a computer, or a laptop or an iPad as many carers, especially older carers, had no access to those devices. Instead, they preferred to use hardcopy versions of the iSupport manual, which were supplied. Fourth, we changed the sample size calculation from the 90% power to detect the intervention effectiveness to 80% power. The latter ensured a minimal sample size in the RCT. We gained approval from the Southern Adelaide Clinical Human Research Ethics Committee (No. 2021/HRE00273) for all changes.

## 3 RESULTS

Between July 1, 2022, and December 31, 2023, we assessed 830 potential carer participants for eligibility. Of those, 530 did not meet the inclusion criteria, and 151 did not return consent forms to the researcher. In total, 149 carers enrolled in the study, completed the baseline survey, and were randomly assigned to the usual care group (*n* = 77) or the intervention group (*n* = 72). We completed the 6‐month follow‐up by June 30, 2024 and the 12‐month follow‐up by December 31, 2024. Of the 149 participants, 40 dropped out of the study during the 12‐month follow‐up, and the attrition rate was 26.8%. The attrition rates per trial arm at 6 and 12 months are outlined in Figure 2. The main reasons for drop‐out were nursing home admission or death. The eligibility check, carer enrollment, baseline survey, 6‐month follow‐up, and 12‐month follow up figures are outlined in the “Flowchart of the virtual iSupport program” (see Figure 2).

Carers and PLWD in the intervention group and the usual care group showed similar baseline characteristics (see Table 1). No significant differences were observed between the intervention and usual care groups (all *p* >  0.05).

**TABLE 1:** Baseline characteristics of carers and people living with dementia (PLWD) and outcome variables.

Parameter	Total sample *n* = 149	iSupport program *n *= 72	Usual care n = 77
A. Carers			
Gender: *n* (%)			
Male	38 (25.5)	20 (27.8)	18 (23.4)
Female	111 (74.5)	52 (72.2)	59 (76.6)
Age (years): mean (SD)	66.0 (12.1)	65.0 (12.7)	66.9 (11.5)
Relationship with patient: *n* (%)			
Spouse	80 (53.7)	38 (52.8)	42 (54.5)
Non‐spouse	69 (46.3)	34 (47.2)	35 (45.5)
Duration of carer role (years): Median (IQR)	3.0 (1.6‐5.0)	3.0 (1.7‐6.0)	3.0 (1.5‐5.0)
Marital status: *n* (%)			
Married	114 (76.5)	52 (72.2)	62 (80.5)
Unmarried/divorced/widowed	35 (23.5)	20 (27.8)	15 (19.5)
Live in the same house: *n* (%)			
Yes	104 (69.8)	19 (26.4)	26 (33.8)
No	45 (30.2)	53 (73.6)	51 (66.2)
Religion: *n* (%)			
Yes	55 (36.9)	29 (40.3)	26 (33.8)
No	92 (61.7)	42 (58.3)	50 (64.9)
Ethnicity: *n* (%)			
Non‐CALD	119 (79.9)	62 (86.1)	57 (74)
CALD	23 (15.4)	6 (8.3)	17 (22.1)
Employment status: *n* (%)			
Employed	61 (40.9)	30 (41.7)	31 (40.3)
Unemployed/retired	88 (59.1)	42 (58.3)	46 (59.7)
Education level: *n* (%)			
High school and below	38 (25.5)	16 (22.2)	22 (28.6)
Above high school	111 (74.5)	56 (77.8)	55 (71.4)
Financial pressure: *n* (%)			
Yes	74 (49.7)	41 (56.9)	33 (42.9)
No	74 (49.7)	30 (41.7)	44 (57.1)
Hours on care weekly: Median (IQR)	28.0 (10.5‐72.0)	35.0 (12.5‐70.0)	28.0 (10.0‐84.0)
Family members’ help: *n* (%)			
Substantial help	27 (18.1)	11 (15.3)	16 (20.8)
Casual help	69 (46.3)	35 (48.6)	34 (44.2)
No help	45 (30.2)	23 (31.9)	22 (28.6)
Comorbidities: *n* (%)			
Yes	105 (70.4)	50 (70.9)	54 (70.2)
No	43 (28.9)	20 (27.8)	23 (29.9)
SF‐12 mental‐health QoL, mean (SD) (range 0‐100)	40.2 (12.3)	38.7(12.3)	41.5 (12.3
SF‐12 physical‐health QoL, mean (SD) (range 0–100)	47.2 (13.8)	46.1 (14.7)	48.1 (12.9)
RSCSE respite, mean (SD) (range 0–100)	46.0 (27.4)	45.4 (26.2)	46.5 (28.6)
RSCSE behaviors, mean (SD) (range 0–100)	70.6 (17.7)	71.0 (17.1)	70.3 (18.3)
RSCSE upsetting, mean (SD) (range 0–100)	59.0 (20.9)	58.9 (20.0)	59.1 (21.9)
COPE Index‐QS, mean (SD) (range 1–4)	2.6 (0.7)	2.5 (0.6)	2.7 (0.7)
RMBPC Reactions Frequency, mean SD (range 0–4)	1.5 (0.7)	1.5 (0.6)	1.4 (0.7)
B. PLWD			
Gender: *n* (%)			
Male	75 (50.3)	34 (47.2)	41 (53.2)
Female	74 (49.7)	38 (52.8)	36 (46.8)
Age (years), mean (SD)	80.3 (8.4)	78.8 (8.7)	81.7 (7.9)
Religion: *n* (%)			
Yes	68 (45.6)	34 (47.2)	34 (44.2)
No	78 (52.3)	36 (50.0)	42 (54.5)
Ethnicity: *n* (%)			
Non‐CALD	112 (81.9)	61 (84.7)	61 (79.2)
CALD	22 (14.8)	7 (9.7)	15 (19.5)
Education level: *n* (%)			
High school and below	81 (54.4)	53 (73.6)	59 (76.6)
Above high school	66 (44.3)	18 (25.0)	17 (22.1)
Type of dementia: *n* (%)			
Alzheimer's disease	68 (45.6)	34 (47.2)	34 (44.2)
Other types	64 (43.0)	30 (41.7)	34 (44.2)
Probably dementia	17 (11.4)	8 (11.1)	9 (11.7)
Stages of dementia^A:^ *n* (%)			
Mild	87 (58.4)	41 (56.9)	46 (59.7)
Moderate	62 (41.6)	31 (43.1)	31 (40.3)
Comorbidities: *n* (%)			
Yes	122 (81.9)	60 (83.3)	62 (80.5)
No	27 (18.1)	12 (16.7)	15 (19.5)
RMBPC Behavior: mean (SD) (range 0‐4)	1.7 (0.6)	1.7 (0.6)	1.6 (0.6)
QOL‐AD‐Proxy: mean (SD) (range 1‐52)	29.4 (5.2)	28.9 (5.5)	29.8 (5.0)
Hospital admission: no. (%)	102 (68.5)	60 (83.3)	42 (54.5)
Emergency department use: no. (%)	24 (16.1)	14 (19.4)	10 (13.0)

*Note*: Substantial help meant providing 24h or daytime or nighttime care; Mild dementia was based on MMSE score 24‐21; Moderate of dementia was based on MMSE score 10‐20. A higher score on SF‐12 mental‐health QoL indicates better mental‐health‐related QoL; A higher score on SF‐12 physical‐health QoL indicates better physical‐health‐related QoL; A higher score on SF‐12 mental‐health QoL indicates better mental‐health‐related QoL; A higher score on RSCSE respite indicates better self‐efficacy in obtaining respite care; A higher score on RSCSE behaviors indicates better self‐efficacy in responding to changed behaviors of PLWD; A higher score on RSCSE upsetting indicates better self‐efficacy in controlling upsetting thoughts about caregiving; A higher score on COPE Index‐QS indicates better social support; A higher score on RMBPC Behavior indicates more changed behaviors of PLWD; A higher score on RMBPC Reactions Frequency indicates carers’ more distress reactions to the changed behaviors of PLWD; A higher score on QOL‐AD‐Proxy indicates better QoL of PLWD; Hospital admission and emergency department use was the total number of the cohort in a 6 month period.

Abbreviations: CALD, culturally and linguistically diverse; COPE Index‐QS, Quality of Social Support domain in the Older People in Europe Index; IQR, interquartile range; MMSE, Mini‐Mental State Examination; PLWD, people living with dementia; QOL‐AD‐Proxy, Quality of Life in Alzheimer's Disease‐Proxy; RMBPC, Revised Memory and Behavior Problem Checklist; RSCSE, Revised Scale for Caregiving Self‐Efficacy Scale; SF‐12 mental‐health QoL, Short form 12 health survey questionnaire mental component score; SF‐12 physical‐health QoL, Short form 12 health survey questionnaire physical component score.

Carers’ average age was 66 (SD: 12.1) years. The majority were female (*n* = 111, 74.5%), spouse carers (*n* = 80, 53.7%), from the mainstream culture group (*n* = 119, 79.9%), received above high school education (*n* = 111, 74.5%), and lived in the same house with PLWD (*n* = 104, 69.8%). Half of them experienced financial stress. Only 23 carers (15.4%) in the study were from CALD backgrounds; thus, our sample underrepresents CALD carers, who have made up to 32% of the dementia carer population in Australia.^1^ Notably, 105 carers (70.4%) lived with multimorbidity, which was higher than the 50.2% in the older population in Australia.^42^ This may be due to the concentration of older female carers in our study who have higher rates of multimorbidity than their male counterparts in Australia.^42^ This finding underscores the need to support carers to perform self‐care to sustain their carers’ role. Carer participants reported a relatively low SF‐12 QoL MCS mean score of 40.2 (SD: 12.3) and an SF‐12 QoL PCS mean score of 47.2 (SD: 13.8). They also reported a relatively low RSCSE respite mean score of 46.0 (SD: 27.4), a relatively high RSCSE behaviors mean score of 70.6 (SD: 17.7) and an average RSCSE upsetting mean score of 59.0 (SD: 20.9). Their mean score of COPE Index‐QS was 2.6 (SD: 0.7). They reported a relatively low RMBPC Reaction Frequency mean score of 1.5 (SD: 0.7).

Just over half of PLWD were male (*n* = 75, 50.3%) with a mean age of 80.3 (SD: 8.4) years. Most had Alzheimer's disease (*n* = 68, 45.6%) and the remaining had another type of dementia (*n* = 64, 43.0%) or probable dementia (*n* = 17, 11.4%). Most PLWD were from the mainstream cultural group (*n* = 112, 81.9). Only 22 (14.8%) were from the CALD backgrounds; thus, our sample underrepresented PLWD from the CALD background who have made up to 25% of the population living with dementia in Australia.^1^ The majority were in a mild stage of dementia (*n* = 87, 58.4%) and lived with multimorbidity (*n* = 122, 81.9%). They had a relatively low RMBPC Behavior mean score of 1.7 (SD: 0.6). Their QOL‐AD‐Proxy mean score was 29.4 (SD: 5.2). Within a month prior to the baseline, carers reported 17 times of hospital admission (or 11.4%/person/month) and 4 times of emergency department presentation (or 2.7%/person/month) of PLWD.

### 3.1 Intervention effect on primary outcomes

**FIGURE 3:**
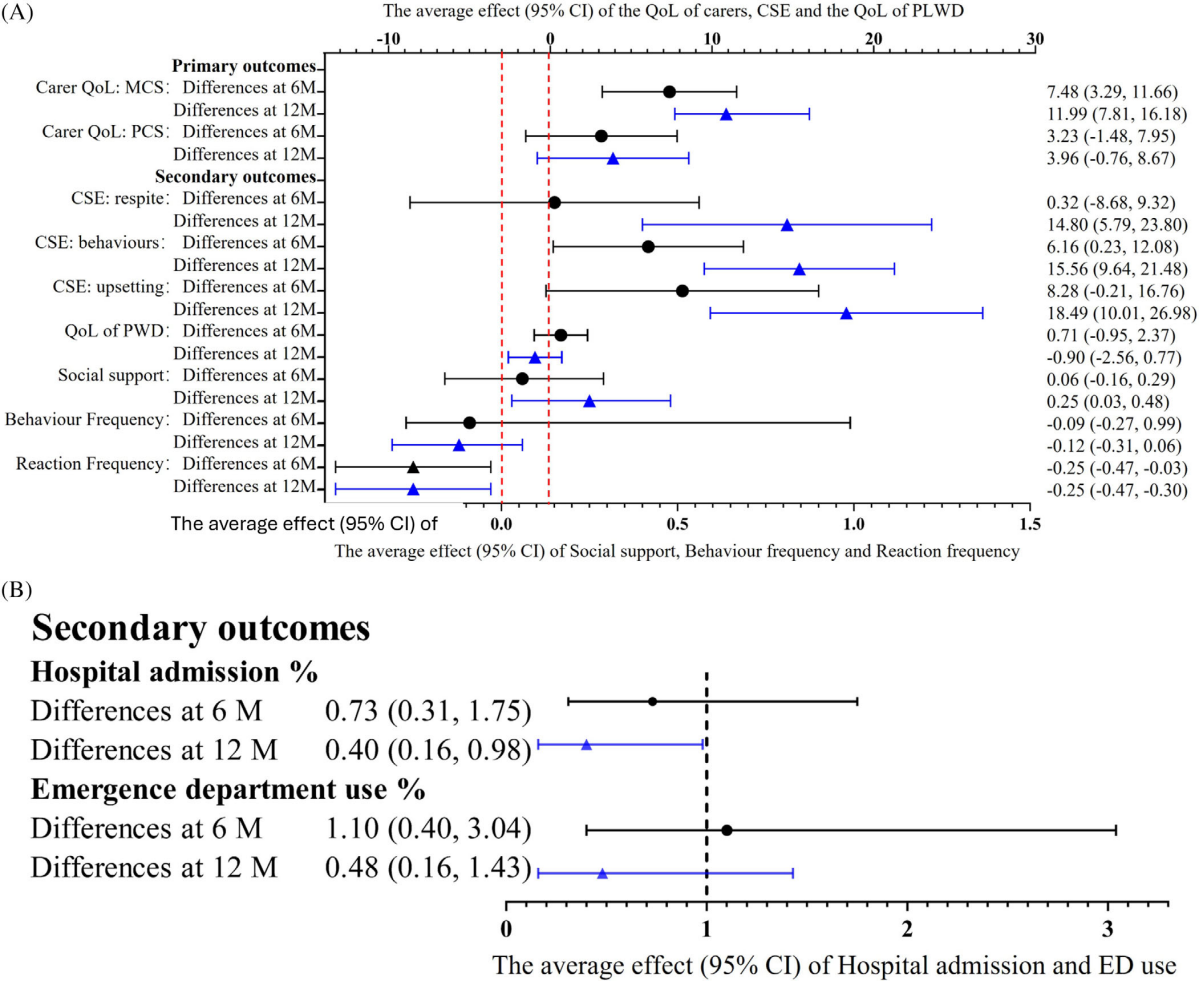
(A) A multivariate mixed effect linear regression model to estimate the intervention effects on the primary and secondary outcomes. All models are adjusted for gender, age, relationship with the care recipients, marital status, duration in care role, live in the same household with PWD or not, religion, employment status, education level, financial pressure, family members’ help and baseline outcome variables. (B) A mixed‐effect Poisson regression model to estimate the intervention effects on the hospital admission rate and the emergency department presentation rate at 6 months and 12 months using the IRR. All models are adjusted for gender, age, relationship with the care recipients, marital status, duration in care role, live in the same household with PWD or not, religion, employment status, education level, financial pressure, family members’ help and baseline outcome variables. CI, confidence interval; CSE = Caregiver self‐efficacy; IRR, incidence rate ratio; M = months; MCS, mental component score; PCS = physical component score; PLWD, people living with dementia; PWD = people with dementia; QoL = quality of life.

We illuminate the changes in outcomes at 6 and 12 months in Figures 3a, Figure 3b and Supplementary file 6, Table S1. Carers in the intervention group reported increased mental‐health‐related QoL points of 7.48 (95% confidence interval [CI]: 3.29, 11.66, *p* < 0.001) at 6 months and 11.99 (95% CI: 7.81, 16.18, *p* < 0.001) at 12 months compared with those in the usual care group. Carers in the intervention group also reported increased physical‐health‐related QoL points of 3.23 (95% CI: ‐1.48, 7.95) at 6 months and 3.96 (95% CI: ‐0.76, 8.67) at 12 months compared with those in the usual care group, but the effect was not statistically significant (*p* > 0.05). PLWD in the intervention group showed increased QoL points of 0.71 (95% CI: ‐0.95, 2.37) at 6 months and decreased QoL points of ‐0.90 (95% CI: ‐2.56, 0.77) at 12 months compared with those in the usual care group, but the effect was not statistically significant (*p* > 0.05).

### 3.2 Intervention effect on secondary outcomes

First, carers in the intervention group reported increased points in RSCSE respite subscale of 0.32 (95% CI: ‐8.68, 9.32) at 6 months and 14.8 (95% CI: 5.79, 23.80) at 12 months compared with those in the usual care group. The effect was not statistically significant at 6 months (*p *> 0.05) but was at 12 months (*p *= 0.001). Second, carers in the intervention group reported increased points in RSCSE behaviors subscale of 6.16 (95% CI: 0.23, 12.08, *p* = 0.042) at 6 months and 15.56 (95% CI: 9.64, 21.48, *p* < 0.001) at 12 months compared with those in the usual care group. Third, carers in the intervention group reported increased points in the RSCSE upsetting thoughts subscale of 8.28 (95% CI: (‐0.21, 16.76) at 6 months and 18.49 (95% CI: 10.01, 26.98) at 12 months compared with those in the usual care group. The effect was not statistically significant at 6 months (*p* > 0.05) but was at 12 months (*p* < 0.001). Fourth, carers in the intervention group reported increased COPE Index‐QS points of 0.06 (95% CI: ‐0.16, 0.29) at 6 months and increased points of 0.25 (95% CI: 0.03, 0.48) at 12 months compared with those in the usual care group. The effect was not statistically significant at 6 months (*p *> 0.05) but was at 12 months (*p *= 0.028). Fifth, PLWD in the intervention group showed decreased RMBPC Behavior Frequency points of ‐0.09 (95% CI: ‐0.27, 0.99) at 6 months and decreased points of ‐0.12 (95% CI: ‐0.31, 0.06) at 12 months compared with those in the usual care group but the effect was not statistically significant (*p *> 0.05). Sixth, carers in the intervention group reported decreased RMBPC Reaction frequency points of ‐0.25 (95% CI: ‐0.47, ‐0.03, *p *= 0.029) at 6 months and decreased points of ‐0.25 (95% CI: ‐0.47, ‐0.30, *p *= 0.026) at 12 months compared with those in the usual care group. Seventh, PLWD in the intervention group showed a 27% lower hospital admission rate (incident rate ratio [IRR] = 0.73, 95% CI: 0.31, 1.75) at 6 months and a 60% lower hospital admission rate (IRR = 0.40, 95% CI: 0.16, 0.98) at 12 months compared with the usual care group. The differences were not statistically significant at 6 months (*p *> 0.05) but was at 12 months (*p *= 0.045). Finally, PLWD in the intervention group showed a 10% higher emergency department presentation rate at 6 months (IRR = 1.10; 95% CI: 0.40, 3.04) but a 50% lower emergency department presentation rate at 12 months (IRR = 0.5; 95% CI: 0.16, 1.43) compared with the usual care group. However, the differences were not statistically significant (*p *> 0.05).

### 3.3 iSupport unit completion and satisfaction with support in the intervention group

Carers completed an average of 7.2 (SD: 3.9) units at 6 months and 7.7 (SD: 3.8) units at 12 months. Their total mean unit completion was 14.8 (SD: 5.3, range: 7–30), and only 15.3% of carers completed 20 units or over, as we recommended to them. Carers showed a very high mean satisfaction score of 4.75 (SD: 0.6) at 6 months and 4.85 (SD: 0.5) at 12 months; thus, it was evident that they were satisfied with the support provided by the trained facilitators in the program. We present the detailed findings in Supplementary file 7, Table S2.

## 4 DISCUSSION

The virtual iSupport Program that incorporated facilitator‐enabled dementia care skills training, peer support, and needs based support in accessing care services demonstrated improved mental‐health‐related QoL (a primary outcome), self‐efficacy in dementia care, social support, and reactions to changed behaviors of PLWD (secondary outcomes) for carers of PLWD. The intervention effects were more evident at the 12‐month follow‐up than those at the 6‐month follow‐up, underscoring the need to have a sufficient intervention duration. To the best of our knowledge, the virtual iSupport Program is the first RCT in pragmatic hospitals and community aged care settings to determine the effectiveness of the program delivered by trained facilitators selected and supervised by site‐specific leaders of the four participating organizations in the study. Therefore, our intervention is more pragmatic^43^ and more likely to be adapted and sustained in these care settings in the implementation phase in the future, compared with other multicomponent interventions delivered by interventionists affiliated with research centers or universities.

The intervention effect on carers’ mental‐health‐related QoL identified in our study concurs with the German REACH II and the United Kingdom (UK) START multicomponent interventions for carers of PLWD delivered in person to individual carers by trained interventionists.^39^, ^44^, ^45^ Our findings support that a virtually delivered multicomponent intervention by trained facilitators can also achieve a similar effect on carers’ QoL. Our findings also align with systematic reviews and meta‐analyses that multicomponent interventions can improve QoL of carers, although these reviews included both virtual and in person interventions.^5^, ^7^ Studies showed consistently that online psychoeducation interventions alone had no effect on QoL of carers.^7^, ^13^, ^21^ This result was confirmed in the recent UK online self‐guided iSupport program intervention (or a single component intervention).^46^ The mechanism underlying the no intervention effect on carers’ QoL was explained as the lack of human contacts to engage carers in the program.^18^, ^46^

We did not detect an intervention effect on carers’ physical‐health‐related QoL, and this finding aligns with the German REACH II trial.^39^ Factors contributing to the physical‐health‐related QoL are related to objective burdens such as time spent on care activities due to their carer responsibilities^47^ which require practical support for carers such as respite care and home care services to achieve physical‐health‐related QoL, especially for older spousal carers.^12^, ^48^ In our study, 53.7% (*n* = 80) of carers were older spousal carers, and 70.4% (*n* = 105) indicated that they lived with two or more chronic conditions, underscoring the need to enable them to use formal care services to relieve their physical burden and improve physical‐related QoL.

We found no intervention effect on QoL of PLWD, and the finding aligns with the UK START delivered in person^44^ and the United States of America (USA) Care Ecosystem program delivered virtually.^49^ Notably, the USA Care Ecosystem program reported improved QoL of PLWD in years 2–5 when the intervention group continued to receive the program based on their needs,^26^ evidencing that needs‐based ongoing support for carers is imperative. A systematic review and meta‐analyses confirm that interventions on carers can improve the QoL of PLWD only if the intervention includes the component of care coordination/case management for PLWD.^6^ Such an intervention component was included in the USA Care Ecosystem program where an unlicensed team navigator acted as the coordinator for both PLWD and their carers in a multidisciplinary team.^26^, ^49^ However, the iSupport facilitators in our program only acted as a coordinator for carers rather than their care recipients, which might have limited the intervention effect on PLWD.

In our study, carers showed improved self‐efficacy in only one of three domains at 6 months but all domains at 12 months post‐baseline, evidencing that a 12‐month intervention duration is needed in this virtual multicomponent intervention. Our findings align with the two virtual multicomponent intervention programs in the USA, although the scales used to measure the self‐efficacy differed in these programs.^19^, ^49^ Self‐efficacy development theory suggests that creating a supportive learning environment for individuals to interact with peers and/or facilitators are conditions for them to experience cognitive, motivational, affective, and behavioral development to believe in their capabilities of accomplishing an area of practice.^50^ Opportunities to interact with others can motivate carers to learn, gain coaching support, and access role‐models by which they are more likely to develop and believe in their capabilities in dementia care.^17^, ^18^, ^20^ Moreover, navigating, accessing, and using care services are well‐recognized challenges facing carers due to structural barriers in the health and social care systems.^9^, ^24^ Thus, having a facilitator or a team navigator to support them is crucial to develop self‐efficacy.^49^, ^51^

We identified that the intervention reduced carers’ distress reactions to the changed behaviors of PLWD, and the finding concurs with other multicomponent interventions delivered in person or virtually.^19^, ^39^, ^49^, ^52^ This intervention effect may be related to carers’ self‐efficacy in responding to changed behaviors of PLWD. Moreover, we also identified that the intervention affected carers’ perceived social support, which was also reported in the German REACH II intervention.^39^ Carers in our program were satisfied with the support they received in the program, including social support which was consistent with their perceived improved social support. However, we did not find that intervention reduced the changed behaviors of PLWD as reported in the German REACH II intervention delivered in person for individual carers.^39^ This finding underscores the need to provide individualized interventions via partnership with carers as reported in two systematic reviews and meta‐analyses.^25^, ^53^

Our intervention demonstrated an effect to reduce hospital admissions by 60% at 12 months post‐baseline. Our finding differs from the USA Care Ecosystem program which reported no intervention effects on hospital admission. The peer support component in our program which was not included in the Care Ecosystem program may have a role to play in reducing hospital admissions but this finding needs to be further confirmed in process evaluation in future studies. In addition, the baseline hospital admission rate of 11.4%/per person/month (or 1.37%/person/year as estimated) in our sample was higher than the hospital admission rate of 37% to 1.26%/person‐year reported in a systematic review and meta‐analyses.^54^ This may be due to the recruitment of PLWD from two tertiary hospitals in our study and a high proportion of PLWD living with multimorbidity in our study, underscoring the need to have a trained facilitator to prepare carers to reduce unplanned hospital admission of PLWD.^55^ We did not identify an intervention effect on reducing emergency department presentations. Our finding differs from the USA Care Ecosystem program, which reported an intervention effect in this outcome.^49^ This result may be due to a low baseline emergency department presentation rate (2.7%/person/month) in our sample compared to the 58.0% incidence rates in a 30‑day period reported in a systematic review and meta‐analyses.^56^

Our study had some limitations. First, CALD carers were underrepresented in our study. Factors affecting our engagement with CALD carers may be due to a lack of culturally and linguistically tailored iSupport programs for them, as we used the English iSupport manual, and the facilitators who delivered the intervention did not match CALD carers’ culture and language use. Second, we recruited carers from two tertiary hospitals and two large‐sized aged care organizations, which do not represent the diverse health and social care organizations that provide dementia care services to carers and PLWD in Australia. Therefore, the study design may have limited the generalizability to care settings that differ from those participating organizations in our study, such as primary care, private care, and small‐ and medium‐sized health and social care organizations. Third, although our trial demonstrated improved mental‐health‐related quality of life for carers, we did not conduct a formal mediation analysis to identify which specific components of the intervention drove this effect. Future research should embed real‐time process evaluations and prespecified mediation frameworks, with sufficient sample size, to isolate the active ingredients responsible for QoL gains.

In conclusion, the virtually delivered iSupport Program demonstrated intervention effectiveness in improving carers’ mental‐health‐related QoL, self‐efficacy, social support, and distress reactions to changed behaviors of PLWD. Our intervention effects on carers align with two previous virtual multicomponent intervention studies.^19^, ^49^ Our program can also achieve the benefits for carers reported in the in‐person multicomponent interventions,^39^, ^44^ and we demonstrate a more pragmatic intervention design to facilitate the adaptation of the intervention compared with these previous interventions. Our study has implications for policymakers to consider policy development to enable health and aged care organizations to adapt or adopt this evidence‐based program to their care settings. Future program adaptation needs to focus on how to integrate the facilitator's role in health and aged care organizations with or without additional funding requirements. Moreover, a standardized training program for facilitators from a variety of vocational backgrounds would need to be available via education and training providers to sustain the program implementation. Additionally, the program needs to be tailored to carers’ culture and language use to engage CALD carers in the program.

## CONFLICT OF INTEREST STATEMENT

All authors have no conflicts to disclose. Author disclosures are available in the supporting information.

## CONSENT STATEMENT

We gained written consent from each carer and their care recipient prior to the commencement of the study. Our site‐specific researchers met the carer and their care recipient in person or online based on their preference to explain the study to them and take informed consent or third‐party consent if the care recipient was unable to take consent themselves. The researchers performed the cognitive assessment for the care recipient using a validated assessment scale to decide if they can take informed consent.

## Supporting information



Supporting Information

Supporting Information

Supporting Information

Supporting Information

Supporting Information

Supporting Information

Supporting Information

Supporting Information

Supporting Information
