# Supplementary material for: Effects of a virtual iSupport Program on carers and people with dementia
Source: Alzheimers Dement. 2025 Sep 29;21(10):e70747. doi: 10.1002/alz.70747 (PMC12479211; doi:10.1002/alz.70747)
Supplement: Supplementary file 1 — Supporting Information [file ALZ-21-e70747-s003.docx]

**SUPPLEMENT**

List of Contents

Supplementary file 1 Outline of iSupport modules and units.

Supplementary file 2 CONSORT reporting checklist

Supplementary file 3 A updated study protocol

Supplementary file 4 The training and support for facilitators

Supplementary file 5 A meeting agenda for peer support meetings

Supplementary file 6 Table S1 Changes in outcomes at 6 months and 12 months

Supplementary file 7 Table S2 iSupport unit completion and satisfaction with the support
