# Supplementary material for: Effects of a virtual iSupport Program on carers and people with dementia
Source: Alzheimers Dement. 2025 Sep 29;21(10):e70747. doi: 10.1002/alz.70747 (PMC12479211; doi:10.1002/alz.70747)
Supplement: Supplementary file 2 — Supporting Information [file ALZ-21-e70747-s006.docx]

**Supplementary file 1: Outline of iSupport modules and units**

| Modules | Units |
| --- | --- |
| 1. Introduction to dementia | 1. What is dementia? |
| 2. Being a carer | 1. The journey together  2. Improving communication  3. Supported decision-making  4. Involving others |
| 3. Caring for yourself | 1. Reducing stress in everyday life  2. Making time for pleasant activities  3. Thinking differently |
| 4. Providing everyday care | 1. Eating and drinking-more pleasant mealtimes  2. Eating, drinking and preventing health problems  3. Toileting and continence care  4. Personal care  5. An enjoyable day |
| 5. Dealing with changed behaviour | 1. Introduction to behaviour changes  2. Memory loss  3. Aggression  4. Depression, anxiety and apathy  5. Difficulty sleeping  6. Delusions and hallucinations  7. Repetitive behaviour  8. Walking and getting lost  9. Changes in judgement  10. Putting it all together |
| 6. Consumer-directed aged care and dementia care | 1. Making informed choices in dementia care  2. Services that are available for younger onset dementia  3. Carer support  4. Dementia Behaviour Management Advisory Service  5. Home care packages  6. Relinquishing the carer role |

Note: Module 6 is developed to suit the local context in Australia.
