# Supplementary material for: Effects of a virtual iSupport Program on carers and people with dementia
Source: Alzheimers Dement. 2025 Sep 29;21(10):e70747. doi: 10.1002/alz.70747 (PMC12479211; doi:10.1002/alz.70747)
Supplement: Supplementary file 4 — Supporting Information [file ALZ-21-e70747-s009.pdf]

Supplementary file 3: A updated study protocol

Protocol

The protocol (project description) is to be submitted with the HREA form to the Southern Adelaide Clinical Human Research Ethics Committee (SAC HREC)

The HREA form ensures your project complies with the ethical considerations for research, outlined in the National Statement on the Ethical Conduct in Human Research.

The protocol provides the SAC HREC with the design, objectives, methodology and rationale on how the research project will be conducted.

How to use this document:

Please treat this as a piece of academic writing, taking into careful consideration readability, spelling and grammar.

Fill out all sections of this form in a clear and concise manner, so anyone reading your application will be able to understand what your research project involves.

If you are copying from another document and pasting into this template, please click Keep text only (T).

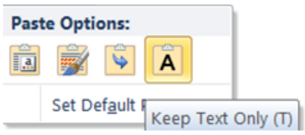

Please ensure you add a version number and date in the footer of this document

HREA submission guidelines are available on the Office for Research website.

**Project Title**: Creating 'partnerships in iSupport program' to optimise carers' impact on dementia care

Project team

The person listed as the Chief Investigator / Principal Investigator is responsible for the conduct of the research and listed study staff until completion of the project.

A student cannot be listed as the Coordinating Principal Investigator or Principal Investigator

Explain the role in the study that each Investigator will perform at each site and clearly state whether Investigators will work on or off the relevant public LHN site(s).

Name: Professor Lily Xiao
Institutional affiliation: College of Nursing and Health Sciences, Flinders University
What is the position of this person on the research project? Coordinating Principal Investigator(CIA)
What are the research activities this person will be responsible for: Professor Xiao is the Principal Investigator (PI) and will take responsibility for the overall governance,

organisation and management of this proposed study. She will be responsible for the conduct of the study at all sites, and for ensuring all annual reviews, amendments and safety reporting. She will play a leadership role in engaging stakeholders including carers and aged care service providers in the study. She will supervise personnel employed in the study. She will also lead the design and implementation of training activities for them to comply with ethics requirements and to adhere to study protocol.<br>Does this person have a current Good Clinical Practice certificate? ☒ Yes / ☐ No
Department and department address: College of Nursing & Health Science, Sturt Road, Bedford Park South Australia 5042.

Contact details: a Health or University email address is preferred<br>☒ I am the contact person for this project	Phone: +61 8 82013419<br>Email: lily.xiao@flinders.edu.au

Name: Ms Langduo Chen
Institutional affiliation: Unit manager for Rehabilitation Ward, Southern Adelaide Local Health Network (SALHN)
What is the position of this person on the research project? Co- Principal Investigator (CIE)
What are the research activities this person will be responsible for: Ms Chen is appointed as a Co-PI as the PI, Professor Lily Xiao, is not a SALHN employee. She will take responsibility for the management of this study within the SALHN. She will also supervise researchers to recruit participants and conduct data collection at SALHN.<br>Does this person have a current Good Clinical Practice certificate? ☐ Yes / ☒ No
Department and department address: Tobruk Rehabilitation Ward Level 5, RAP Building Flinders Medical Centre 1 Flinders Drive, Bedford Park 5042

Contact details: a Health or FUSA email address must be used<br>☐ I am the contact person for this project	Phone: 08 84042062<br>Email: langduo.chen@flinders.edu.au

Name: Professor Julie Ratcliffe

Institutional affiliation: Professor of Health Economics and Mathew Flinders Fellow, Health and Social Care Economics Group, Caring Futures Institute, Flinders University. Honorary Professor, School of Health and Related Research, University of Sheffield. Honorary Professor, Institute of Health and Wellbeing, University of Glasgow
What is the position of this person on the research project? Chief Investigator(CIB)
What are the research activities this person will be responsible for: Professor Ratcliffe be responsible for the design and delivery of the cost-effectiveness and budget impact analyses. She will supervise health the economist employed in the project and support site-specific researchers in data collection and data entry regarding cost-effective and budget impact analyses in the project.<br>Does this person have a current Good Clinical Practice certificate? ☐ Yes / ☒ No
Department and department address: College of Nursing & Health Science<br>Sturt Road, Bedford Park South Australia 5042, GPO Box 2100 Adelaide SA 5001

Contact details: a Health or University email address must be used<br>☐ I am the contact person for this project	Phone: +61 8 82013702<br>Email: julie.ratcliffe@flinders.edu.au

Name: Dr Claudia Meyer
Institutional affiliation: Research Fellow, Bolton Clarke Research Institute, Melbourne, Australia.
What is the position of this person on the research project? Chief Investigator (CIC)
What are the research activities this person will be responsible for: Dr Meyer will take responsibility for the management of this study within Bolton Clarke Victoria. She will supervise the site-specific research assistant at Bolton Clarke Research Institute to work in the study. She will also assist the recruitment of program facilitator and informal carers to participate in the Australian iSupport program.<br>Does this person have a current Good Clinical Practice certificate? ☒ Yes / ☐ No
Department and department address: Level 1, 347 Burwood Highway Forest Hill, VIC 3131

Contact details: a Health or FUSA email address must be used<br>☐ I am the contact person for this project	Phone: 03 8531 2500<br>Email: cmeyer@boltonclarke.com.au

Name: Dr Michael Chapman	
Institutional affiliation: Director, Palliative Care at Canberra Health Services, ACT	
What is the position of this person on the research project? Chief Investigator (CID)	
What are the research activities this person will be responsible for: Dr Chapman will take responsibility for the management of this study within the Canberra Health Services. He will supervise the research assistant at Canberra Health Services to work in the study. He will also assist the recruitment of program facilitator and informal carers to participate in the Australian iSupport program.<br>Does this person have a current Good Clinical Practice certificate? ☒ Yes / ☐ No	
Department and department address: Building 19 Level 5, Palliative Care, Canberra Hospital, ACT, 2606.	
Contact details: a Health or FUSA email address must be used<br>☐ I am the contact person for this project	Phone: (02) 51247693<br>Email: Michael.Chapman@act.gov.au

Name: Dr Shahid Ullah
Institutional affiliation: Senior Lecture in Biostatistics at the College of Medicine and Public Health (CMPH), Flinders University.
What is the position of this person on the research project? Chief Investigator (CIF)
What are the research activities this person will be responsible for: Dr Ullah brings extensive biostatistical skills to the project team. He will supervise research assistant across study sites to randomly assign participants into the intervention group and usual care group, data collection and data analysis for the planned randomised controlled trial. He will contribute to the reporting of findings, particularly from a biostatistics perspective.<br>Does this person have a current Good Clinical Practice certificate? ☐ Yes / ☒ No

Department and department address: College of Medicine and Public Health (CMPH)Sturt Road, Bedford Park South Australia 5042

Contact details: a Health or University email address must be used<br>☐ I am the contact person for this project	Phone: +61 8 82012341<br>Email: shahid.ullah@flinders.edu.au

Name: Professor Alison Kitson
Institutional affiliation: College of Nursing and Health Sciences, Flinders University
What is the position of this person on the research project? Chief Investigator (CIG)
What are the research activities this person will be responsible for: Professor Alison Kitson is an internationally recognised knowledge translation expert. She will be responsible for the design and implementation of the study. She will oversee the program facilitation and the analysis of enablers and barriers to the implementation of the iSupport program throughout the trial.<br>Does this person have a current Good Clinical Practice certificate? ☐ Yes / ☒ No
Department and department address: College of Nursing and Health Sciences, Sturt Road, Bedford Park South Australia 5042 GPO Box 2100 Adelaide SA 5001

Contact details: a Health or University email address must be used<br>☐ I am the contact person for this project	Phone: 08 82013492<br>Email: alison.kitson@flinders.edu.au

Name: Dr Andre Queiroz De Andrade
Institutional affiliation: Senior Research Fellow, Digital Health, University of South Australia
What is the position of this person on the research project? Chief Investigator (CIH)
What are the research activities this person will be responsible for: Dr. Andrade will participate in all aspects of the proposed study and will lead the technology aspects of the intervention using digital technology and the carer-directed technologies to enhance the intervention<br>Does this person have a current Good Clinical Practice certificate? ☒ Yes / ☐ No

Department and department address: Quality Use of Medicines and Pharmacy Research Centre<br>UniSA Clinical and Medical Sciences, University of South Australia<br>GPO Box 2471 Adelaide SA 5001

Contact details: a Health or FUSA email address must be used<br>☐ I am the contact person for this project	Phone: +61 8 8302 0571<br>Email: Andre.Andrade@unisa.edu.au

Name: Dr Craig Whitehead
Institutional affiliation: Regional Clinical Director for Rehabilitation and Aged Care, Southern Adelaide Local Health Network (SALHN)
What is the position of this person on the research project? Investigator
What are the research activities this person will be responsible for: Dr Whitehead will take responsibility for the management of this study within the SALHN. He will also be responsible for multidisciplinary clinicians' engagement in the iSupport program by recruiting dementia care experts to the Advisory Group. He will also assist the recruitment of trained health professionals and informal carers to participate in the iSupport program.<br>Does this person have a current Good Clinical Practice certificate? ☐ Yes / ☒ No
Department and department address: Division of Rehab, Aged Care & Palliative Care, Rehabilitation & Palliative Care Building, Level 4, Flinders Medical Centre, Flinders Drive, BEDFORD PARK SA 5042

Contact details: a Health or FUSA email address must be used<br>☐ I am the contact person for this project	Phone: 8404 2440<br>Email: Craig.Whitehead@sa.gov.au

Name: Sue McKechnie
Institutional affiliation: Executive Manager Community Services, Resthaven Incorporated.
What is the position of this person on the research project? Investigator

What are the research activities this person will be responsible for: Ms McKechnie will take responsibility for the management of this study within Resthaven. She will assist recruitment of informal carers and dementia experts to the Advisory Group from Resthaven and via the Aged & Community Services Australia platform. She will also assist the recruitment of trained health professionals and informal carers to participate in the Australian iSupport program.<br>Does this person have a current Good Clinical Practice certificate? ☐ Yes / ☒ No
Department and department address: 6 Bartley Crescent Wayville South Australia 5034

Contact details: a Health or FUSA email address must be used<br>☐ I am the contact person for this project	Phone: (08) 8373 9058<br>Email: sue.mckechnie@resthaven.asn.au

Name: Dr. Rachel Milte
Institutional affiliation: Senior Research Fellow, Caring Futures Institute, College of Nursing and Health Sciences
What is the position of this person on the research project? Investigator
What are the research activities this person will be responsible for: Dr Milte will participate as an integral member of the research team for all stages of the project. She will be responsible for the design and delivery of the cost effectiveness and budget impact analyses.<br>Does this person have a current Good Clinical Practice certificate? ☐ Yes / ☒ No
Department and department address: College of Nursing and Health Sciences, Sturt Rd, Bedford Park, SA 5042

Contact details: a Health or FUSA email address must be used<br>☐ I am the contact person for this project	Phone: 61 8 8201 3088<br>Email: rachel.milte@flinders.edu.au

Name: Ying Yu
Institutional affiliation: College of Nursing and Health Sciences

What is the position of this person on the research project? PhD student, investigator
What are the research activities this person will be responsible for: Ms Ying Yu is a PhD candidate and is supervised by Professor Xiao, Dr Ullah and Dr Meyer in the project. She will participate in the whole project. More specifically, Ms Yu will assist the Chief investigator, Professor Xiao, to coordinate the project across all trial sites, recruit participants, conduct data collection at SALHN and Resthaven. She will also work with Professor Xiao, Dr Ullah and Dr Meyer to analyse quantitative and qualitative data collected from all trial sites and report findings.<br>Does this person have a current Good Clinical Practice certificate? ☒ Yes / ☐ No
Department and department address: College of Nursing and Health Sciences, Sturt Rd, Bedford Park, SA 5042

Contact details: a Health or FUSA email address must be used<br>☐ I am the contact person for this project	Phone: 0404383505<br>Email: ying.yu@flinders.edu.au

Resources

**What resources are necessary for the project to be conducted**?<br>These resource types will need to be taken into consideration including the funding to support the project, the clinics or community aged care centres to provide participations, human resources (the research team) to conduct the project, material resources and supplies (software for data analysis, cabin to store the documents, etc), the coordination and cooperation with other institutes<br>This study is funded by the Australian Government via 2020 Medical Research Future Fund (MRFF) 2020 Dementia Ageing and Aged Care Mission and 2020 NHMRC/DCRC World Class Research Project Grants. The funding will assist Ms Ying Yu to work as a full-time PhD student and other personnel to work in the project to achieve the study objectives. The project is fully supported by four industry partners including Southern Adelaide Local Health Network (SALHN; led by Dr Craig Whitehead and Ms Langduo Chen), Canberra Health Services (led by Dr Michael Chapman), Bolton Clarke Victoria (led by Dr Claudia Meyer) and Resthaven SA (led by Sue McKechnie) via their signed agreements on the 2020 Medical Research Future Fund (MRFF) and 2020 NHMRC/DCRC World Class Research Project Grants (available on the request). The

project team possesses expertise, resources and collaboration with industry partners to implement research activities described in this study protocol.
**Please declare what funding support and amount is being sought or has been secured for this project**:<br>This study is part of large project funded by the Australian Government via 2020 Medical Research Future Fund (MRFF) 2020 Dementia Ageing and Aged Care Mission ($1,406,658) and 2020 NHMRC/DCRC World Class Research Project Grants ($536,586).

Project design

Please refer to the National Statement Chapter 3.1 Elements of Research for guidance on to how to ensure this research is conducted in line with core ethical principles.

**Introduction –** Please provide a brief overview of the study:<br>This study is part of a large health service research project: "Creating 'Partnership in iSupport program' to optimise carers' impact on dementia care". The 'Partnership in iSupport program' includes (1) a program facilitator who acts as a link worker to assist informal carers (family, friends and neighbours) to navigate, access and utilise dementia care services; (2) psychoeducation for informal carers to improve their dementia care capabilities; and (3) virtual carer support groups to strengthen social support for informal carers (we use the word 'carers' onwards in the protocol). The aims of the project are to (1) co-design activities delivered by iSupport Program facilitators (Phase 1); (2) determine the intervention efficacy and cost-effectiveness (Phase 2); and (3) translate the program into practice (Phase 3). In this application, we seek ethics approval for Phase 2: determine the intervention efficacy and cost-effectiveness of the 'Partnership in iSupport program'.<br>The overall aim of this study is to improve quality of life for people living with dementia (PLWD) and their carers by creating an innovative patient-centred approach, 'Partnership in iSupport program', which supports holistic and multidisciplinary care for PLWD.
**Background and literature review**<br>In Australia, 200,000 informal carers provide care for PLWD in the community (Alzheimer's Australia, 2015). They are the cornerstone of helping PLWD remain at home for as long as possible (Cheng et al., 2020). However, they have received less education preparation and limited ongoing support than professional carers to manage

dementia and other complex health issues for PLWD (Bressan, Visintini, & Palese, 2020). Care services for the PLWD after diagnosis are highly fragmented and difficult for carers to navigate and utilise (Steiner et al., 2020). Carers feel socially isolated due to time spent on care, stigma and the lack of quality social networks (Greenwood, Mezey, & Smith, 2018). Caregiver stress and distress are widely reported and contribute to poor health and quality of life (QoL) of both carers and PLWD and to the premature permanent admission to nursing home care of PLWD (Cepoiu-Martin, Tam-Tham, Patten, Maxwell, & Hogan, 2016; Stall et al., 2019). A recent study in the USA, using the National Inpatient Sample from 2012 to 2016, revealed that 40% of hospitalisations of PLWD were due to potentially preventable conditions, for example falls, injuries, dehydration, poisoning or uncontrolled chronic diseases (Anderson, Marcantonio, McCarthy, & Herzig, 2020) strongly suggesting that educating and supporting carers to manage dementia and disease progression can avoid emergency department uses and hospital admissions.

We propose a 'Partnership in iSupport program' built on collaboration between dementia care service providers and informal carers of PLWD in hospital and community aged care settings to tackle the current problems. The intervention comprises (1) having a program facilitator appointed by the participating hospitals or the community aged care providers to act as link workers to assist carers to navigate, access and utilise care services to meet the holistic care needs of the PLWD; (2) a psychoeducation program for carers to improve their knowledge and skills in managing dementia using the online Australian iSupport for Dementia; and (3) virtual carer support groups to enhance socialisation and reduce social isolation for carers. Having a link worker who acts as a single point of contact for carers for a minimum of one-year post-diagnosis support is a standard care service in Scotland (Alzheimer Scotland, 2019). This kind of support is viewed as an innovative dementia care model in a research paper submitted to the 2018-2020 Royal Commission into Aged Care Quality and Safety (Dyer et al., 2019). Combining the three components in a program reflects the stakeholders' expectations for providing a one-stop-shop for PLWD after diagnosis in a recent Australia study(Steiner et al., 2020). The program will use digital technology to improve reach to carers.

We have strong evidence through a systematic review by Cheng et al. (2020) that these three intervention components can significantly improve positive thoughts, subjective wellbeing, ability to manage dementia, social support, reduce mental health problems and stress for carers. The review has also confirmed that multicomponent intervention has the largest effects, compared to an intervention with a single component (Cheng et

al., 2020). This is health service research and our strong support from four partner organisations, Resthaven, Bolton Clarke Victoria, Southern Adelaide Local Health Network (SALHN) and Canberra Health Services is critical to this project success. CIA Xiao has recently led a pilot study with 25 carers in collaboration with Resthaven to test the feasibility of this study and has confirmed that the intervention design was widely acceptable by both carers and the care service provider (Xiao, 2020).

A link worker and potential impact: Two recent systematic reviews have confirmed that having a link worker to assist carers to navigate, access and utilise dementia care resources and multidisciplinary care services can significantly improve carers' QoL and reduce their stress (Cheng et al., 2020; Goeman, Renehan, & Koch, 2016). A link worker is essential to bridge care gaps and prevent care crises during transitions between care settings and types (Alzheimer Scotland, 2019).

Psychoeducation and potential impact: Systematic reviews have determined that psychoeducation programs can significantly improve carers' QoL, self-efficacy and reduced stress (Cheng et al., 2020; Frias et al., 2020). The Australian iSupport program is a well-designed psychoeducation program for informal carers and is adapted from a generic version of the World Health Organization (WHO) iSupport for Dementia led by CIA Xiao (Xiao et al., 2020). iSupport embeds holistic care, for example, providing opportunities for PLWD to choose and control activities which are meaningful, enjoyable, and relevant to their life experience. iSupport uses techniques such as cognitive behavioural therapy to help carers develop positive thoughts, problem-solving and coping skills, self-care, and assertiveness when seeking help from relatives, friends and formal care service providers. The program covers dementia care from early stage to the end of life. Therefore, it helps carers learn about changes as dementia progresses and take proactive action to gain timely treatment and care. When appropriate, the program introduces dementia care resources and multidisciplinary care services with weblinks for carers to access. The Australian iSupport program currently contains 6 modules and 30 units: introduction to dementia; being a carer; caring for me; providing everyday care; and a person-centred care approach to changed behaviour and my engagement in consumer directed care.

Virtual carer support groups and potential impact: The systematic review by Cheng et al. has confirmed that carer support groups can significantly reduce stress and improve self-efficacy and QoL for carers (Cheng et al., 2020). Video-streaming meetings facilitated by trained health professionals demonstrates better health

outcomes for carers (Etxeberria, Salaberria, & Gorostiaga). Virtual support groups enable carers to share their knowledge, experiences in dementia care, to learn from role models and to help each other (Etxeberria et al.). Working carers particularly welcome virtual carer support as they can choose a time to participate.

Creating reciprocal partnerships to support patient-centred approach:
As detailed in the project methodology, iSupport program facilitators appointed by care service providers will assist carers to identify the individualised care needs of PLWD in a timely manner, especially when the PLWD's health condition is deteriorating or during the transitions between care settings (i.e., hospital-to-home) and care types (i.e., receiving palliative care at home). The facilitator will also assist carers to access and utilise relevant care services and co-plan care activities with carers and PLWD. It is anticipated that these supports will meet the care needs and improve QoL of PLWD, prevent and manage behavioural and psychological symptoms of dementia (BPSD) and complications; thus, reduce preventable conditions that contribute to emergency department uses and hospitalisations of PLWD. Further, the carer support group activities facilitated by the facilitator in the study will create opportunities and empower carers to provide constructive feedback to care service providers regarding the strengths and weaknesses of service. Their feedback will benefit the continuous quality improvement, organisational development, and staff development for the care service providers. Poor quality of care was identified by the 2018-2020 Royal Commission into Aged Care Quality and Safety as a significant issue. Only 20% of people receiving home care package felt their care needs were always met across all quality-of-care attributes (Ratcliffe et al., 2020). The reciprocal partnerships built through the program will have high potential to mitigate the issues.

Hypothesis

Primary hypotheses: Compared to those allocated to the usual care group, (1) carers receiving the intervention will report (a) at least a 5-point higher mean score on the mental component of the 12-Item Short-Form Health Survey (SF-12) and (b) an improved mean score on the physical component of SF-12; (2) their care recipients will report an improved mean score on the QoL in Alzheimer's Disease (QOL-AD)-Proxy at 12-months follow-up.

Secondary hypotheses: Compared to those allocated to the usual care group, (1) carers in the intervention group will report (a) an improved mean score on the Caregiving Self-Efficacy Scale and (b) an improved mean score on the Quality of Social Support

Scale; (2) their care recipients will report (carers acting as proxy respondents) (a) a reduced mean score on the Revised Memory and Behaviour Problem Checklist and (b) fewer unplanned hospital admissions, less emergency department uses and less use of permanent residential aged care at 12 months; and (3) provision of the intervention will be more cost-effective than existing care.
**Aims** - (1) **Aim 1:** to determine the intervention efficacy; (2) **Aim 2:** to establish the intervention cost-effectiveness; and (3) **Aim 3:** to understand carers' experiences in the program.
**Objectives** - How will investigators achieve the aims of the research project?<br><br>**Objective 1:** A multicentre randomised controlled trial (RCT) with carers of PLWD will be conducted to test the primary and secondary hypotheses or the intervention efficacy (Aim 1). The intervention will last 12 months.<br>**Objective 2:** The cost effectiveness analysis will be undertaken alongside the RCT (Aim 2). The primary outcome will be the incremental cost per quality adjusted life years (QALYs) gained for the intervention compared to usual care.<br>**Objective 3:** A qualitative descriptive design described by Doyle et al. (2019) will be applied to understand carers' experiences in the program (Aim 3) using data from the virtual carer support group meetings.
**Expected outcomes** - What do the investigators anticipate the outcomes of this research will be?<br>1. The expected outcomes for carers include improved quality of life (QoL), improved self-efficacy, improved quality of social support and positive learning experience in the program.<br>2. The expected outcomes for the persons with dementia include improved quality of life (rated by carers) and reduced memory and behaviour problem (rated by carers).<br>3. Improved quality adjusted life years (QALYs) from the perspective of carers and people with dementia compared to usual care.
**Rationale / justification**<br>In Australia, 200,000 informal carers provide care for people with dementia in the community and more than half of them are of working age (Alzheimer's Australia, 2015). They are the cornerstone of helping people with dementia remain at home for as long as possible (Alzheimer's Australia, 2015). They also play a crucial role in preventing dementia associated complications, reducing hospitalisation rates for this population and mediating treatment interventions led by health professionals (Bott et

al., 2019). However, they are less prepared to take on their role than professional carers (Bott et al., 2019; Xiao et al., 2020). Better preparing this large carer cohort through education and peer support requires innovative models of program delivery with strong support from various dementia care service providers. Online education and virtual social support for carers demonstrate efficacy and increase reach to carers (Egan et al., 2018; Etxeberria et al.; Oliver et al., 2017). Dementia care service providers need to involve carers in dementia education to strengthen partnerships with them. The partnership can be critical for achieving person-centred care and effective treatment interventions for people with dementia (Bott et al., 2019; Xiao et al., 2020). We have gained strong support from four industry partners, Southern Adelaide Local Health Network (SALHN), Canberra Health Services, Bolton Clarke Victoria and Resthaven SA, to implement and evaluate the Australian iSupport program in community aged care and hospital memory and geriatric clinics, aged care and dementia care wards, and ambulatory geriatric services, generating rigorous evidence to inform dementia care policy and practice development.

Research project setting

The participants of the study are informal carers of PLWD in home care settings. The interventions will be delivered by four participating organisations in the study including SALHN, Canberra Health Services, Resthaven SA and Bolton Clarke Victoria.

Methodological approach

Study Design

A multicentre RCT with carers of PLWD will be conducted to test the primary and secondary hypotheses. The intervention will last 12 months. The cost effectiveness analysis will be undertaken alongside the RCT. A qualitative descriptive design will be applied to explore carers' experience in the program using data from the virtual carer support group meetings. The across-method triangulation used in the study via a combination of an RCT and a qualitative strand will facilitate a more holistic understanding of iSupport implementation (Denzin, 2017).

Settings and participants

Participants will be informal carers (family, friends and neighbours) of people living with dementia (PLWD) who live at home. They will be recruited through various care settings as described in the following:

(1) SALHN and Canberra Health Services:

The study will be conducted in various care settings managed by SALHN and

Canberra Health Services, for example in the memory and geriatric clinics, ambulatory geriatric services and falls clinics for community dwelling PLWD, aged care and dementia care wards where PLWD are ready to be discharged home and are expected to live at home for at least 12 months.

(2) Resthaven SA and Bolton Clarke Victoria:
The study will be conducted in community aged care settings where PLWD receive home care package and have informal carers to support them at home.

Randomisation and interventions
After baseline data collection, carers will be randomly assigned to receive either the iSupport program or the usual care. To ensure the two groups are of equivalent size and conditions, a block randomisation will be used to allocate carers to one of the two treatment groups for each recruitment site. Stratification will also be used in the block randomisation to ensure equivalent distribution of spouse carers versus non-spouse carers and care recipients with mild versus moderate dementia in each treatment group.
Arm 1–Usual care group: Carers will receive the usual carer support provided by Dementia Australia or other publicly funded carer support. They will receive a monthly reminder email that directs them to the Dementia Australia website where they can seek support if they wish.
Arm 2–Intervention group: The planned interventions are described in the following.

(1) Managing transitions: In each study site, carers will be assigned to an iSupport program facilitator and encouraged to request individualised support from the facilitator during transition between care settings (i.e. from hospital to home transitional care) and types (i.e. receiving ambulatory geriatric services, or rehabilitation or palliative care at home in addition to usual home care package) of the PLWD.
(2) Managing dementia progression: Carers are also encouraged to contact the facilitator to discuss changes in PLWD (i.e., signs of deterioration, changed behaviours and complications) and gain advice to access relevant multidisciplinary care services for timely treatment and care. The facilitator will follow the carers until their needs for support have been met.
(3) Psychoeducation: Carers will select at least 20 out of 30 learning units that are relevant to them from the online iSupport program to learn over the first 6 months of the trial and revisit or learn new units when they feel it is needed in the second 6 months. The expected time spent on learning the online iSupport learning modules

is no more than 12 hours in total. The completion of each unit will be measured through the program design and will be recorded as data for compliance with the intervention. Carers will receive a certificate for each unit they complete, a strategy to motivate them in the program.<br>(4) Carer support group: In each study site, the facilitator will assign the 23 carers into one of two support groups. The facilitator will conduct a monthly online carer support group meeting lasting no more than 30 minutes. Managing transitions between care settings and types and managing disease progression will be standard meeting agenda so that carers can share their experiences and support each other in these dementia caregiving areas. The meetings will be recorded for carers in the same group to access. The facilitator will also create carer support groups in WhatsApp to encourage carers to talk or send text messages to their peers in the same group to strengthen social support. Text message or follow up phone calls will be used if carers cannot access WhatsApp or zoom (i.e., have no internet or smartphone). The facilitator will analyse group interactions, investigate carers' needs for support and provide feedback to them.<br>(5) Feedback on services: The facilitators will collect carers' feedback via regular carer support meetings and discuss the feedback in their organisation's quality improvement meetings.<br>(6) Confidentiality: Carers will follow an agreed protocol as described in the information sheet to maintain confidentiality and privacy in group activities.
**Consumer and Community engagement –** investigators are encouraged to consult with Consumer and Community groups with the design of their research. Please outline any consultation that has occurred.<br>**Governance and consumer engagement:** The research proposal has been reviewed by 4 carers and 3 staff nominated by industry partners in the study. Moreover, the interventions as described above have been discussed with stakeholders (carers and staff who provider care for people with dementia) in Phase 1 prior to this study phase. Their feedback has been addressed to ensure their voice in this study is heard from the outset. A Steering Committee will be established to govern the study. The committee members will include the project team. The committee will oversee all processes and outcomes of the study and will meet monthly video meetings to ensure the rigor of the study is maintained. A PhD student, Ying Yu will be located at Flinders University to coordinate the project across all sites, assist consultations with stakeholders, update the project website and report findings. A Co-design Advisory Group will include 5 carers and 5 professional dementia care experts to ensure

consumer involvement. The researcher, Ying Yu, will coordinate activities with members of the Co-design Advisory Group. The project team will meet quarterly with the Advisory Group via online meetings to foster the co-design of iSupport implementation strategies.
**What are your outcome measures**?<br>**Outcome measures:** All scales to measure outcomes are validated and were tested in the pilot study.<br>**Primary measures:**<br>(1) Carers: SF-12 (Ware, Kosinski, & Keller, 1996): The SF-12 includes 12 items measuring two domains: mental health-related QoL and physical health-related QoL. Construct validity is 0.92 for the mental component and 0.91 for the physical component. The test-retest reliability for the mental component is 0.76 and for the physical component is 0.89. Higher scores indicate better QoL.<br>(2) Care recipients' Quality of Life in Alzheimer's Disease (QOL-AD)-Proxy (Logsdon, Gibbons, McCurry, & Teri, 2002): This scale has 13 items. It has an internal consistency of 0.84-0.86 and test-retest reliability of 0.76-0.92. Higher scores indicate better QoL.<br>**Secondary measures:**<br>(1) Caregiving Self-Efficacy Scale (Steffen, McKibbin, Zeiss, Gallagher-Thompson, & Bandura, 2002): This scale includes 3 subscales with 15 items: self-efficacy for obtaining respite, responding to atypical patient behaviours and controlling upsetting thoughts about caregiving. It shows internal consistency of 0.8 and test-retest reliability of 0.70. Higher scores indicate better self-efficacy.<br>(2) Quality of support (Moholt , Friborg, Skaalvik, & Henriksen, 2018): It includes 5 items and shows an internal consistency of 0.76 and test-retest reliability of 0.80. Higher scores indicate better social support.<br>(3) The Revised Memory and Behaviour Problem Checklist (Teri et al., 1992): It includes 24 items measuring the frequency of memory and behaviour problems of PLWD and carers' reaction to those problems in three subscales: depression, disruption and memory-related problems. It has an internal consistency of 0.84 for frequency of behaviour and 0.90 for reaction to the behaviour and construct validity. Lower scores indicate less frequency of memory and behaviour problems and carers feeling less upset.<br>**Measures for cost-effectiveness:**<br>(1) The primary measure of cost-effectiveness will be the incremental gains in QALYs from the perspective of carers and people with dementia compared to usual care.

Resources associated with the development and implementation of the intervention will be documented and costed according to established best practice guidelines (Drummond et al., 2015). SF-12 responses between baseline and 12 months will be converted into health state utilities for the calculation of QALYs for carers using the SF-6D preference-based scoring algorithm developed by Brazier et al. (Brazier, Ratcliffe, Salomon J, & Tsuchiya, 2017). QOL-AD-Proxy responses will be similarly converted using the preference-based scoring algorithm developed by Comans et al. (Comans et al., 2020).

(2) Linked administrative health data from Data Linkage Services, including Pharmaceutical Benefits Scheme (PBS) and Medicare Benefits Schedule (MBS) utilisation, and hospital and emergency department uses, will be used to capture health service use for PLWD and their carers for 12 months prior and 12 months post recruitment with permission of the carers and their care recipients (when appropriate). Pre-baseline resource use data will provide additional baseline variables to control for potential confounding. The difference between pre- and post-baseline use of health services will be used to measure changes in PLWD's levels of utilisation and cost of health services in both study arms.

(3) Data on health and social care visits outside those provided by MBS will be collected from participants using the resource utilisation in dementia (RUD) questionnaire (Wimo, Wetterholm, Mastey, & Winblad, 1998).

(4) Unit costs will be derived from hospital finance departments and Australian Refined Diagnosis Related Groups cost weights. Cost of nursing home care will be calculated using basic daily and accommodation fees apportioned according to length of stay.

Carers' experiences in the iSupport for Dementia program:

Carers' experiences in intervention program will be explored by analysing audio-recorded monthly Zoom video meetings with the program facilitators and their peers in the intervention group. The messages (text messages via mobile, email and WhatsApp, request for support) and time spent by facilitator to provide the support will be documented and analysed. Facilitators will document their assessment of carers' needs, actions to meet carers' needs and outcomes in a structured facilitator portfolio (see attachment: Facilitator portfolio) and submit to the project team as part of date for analysing carers' experience in the trial. Facilitators will use code for study site and pseudonyms for carers in the portfolios to maintain confidentiality of information for carers.   A descriptive qualitative study design using thematic analysis will be applied

to achieve this study objective. Findings will be reported as themes that represent carers' learning experiences.
**Project duration**:<br>The intervention will last 12 months. To include time for participants recruitment, we anticipate this study will last 18-24 months.

Participant selection and activities

Explain how participants will be recruited or how their data will be selected (e.g. for a registry).

Describe sources and methods that will be employed in the identification and recruitment/selection of potential participants (e.g., clinics, referring doctors, adverts, and time periods) or of historical data (e.g. medical records, databases).

You should make a distinction between how you will recruit/select control participants compared to other groups if performing a comparative intervention.

**How many participants will be selected for the study**?<br>Based on sample size calculation, 184 carers in total. We plan to recruit 46 carers (23 in each arm) in each of the 4 study sites.
**How are they identified as possible participants?**<br>**SALHN and Canberra Health Services:**<br>Based on inclusion and exclusion criteria, carer participants will be identified by clinicians from various care settings, for example in the memory and geriatric clinics, aged care and dementia care wards, ambulatory geriatric services and falls clinic. We will also publish recruitment flyer on social media to recruit participants via these recruitment sites.<br>**Bolton Clarke Victoria and Resthaven SA:**<br>Staff in community aged care settings will identify potential participants by checking the database of their clients.<br>**Community**<br>1) Contact the local council to distribute recruitment flyers to their support/activity groups (i.e., but not limited to Mitcham council, Onkaparinga council); 2) Put up flyers on social media (i.e., Facebook and Twitter); 3) Put up flyers on the notice board in the community (i.e., library and supermarket notice board); 4) Distribute flyers to local church group;5) Distribute flyers to super GP clinic; 6) Contact Canberra Health Services' collaborative organisations and community organisations in Canberra and surrounding regions (i.e., but not limited to Dementia Australia, CarersACT, Health

Care Consumers Association, ACT Disability Aged and Carer Advocacy Service (ADACAS), and Multicultural Association of Canberra) to disseminate study details (e.g., via newsletters, posters, presentations). 7) Disseminate study details to care settings outside of Canberra Health Services in Canberra and surrounding regions (e.g., private geriatric clinics in Canberra)

Pre-screen for eligibility – waiver of consent

The recruitment method must be compliant with the Health Care Act 2008. If you need to access a patient's medical records to pre-screen for eligible participants, and you do not have prior patient consent to do so or are not part of the patient's clinical care team, you will need to apply for an exemption under 93(3)(f).

Are you requesting a waiver of consent to pre-screen?

☐ No

☒ Yes

Under s93(3),(f) of the Health Care Act 2008, we wish to apply for an exemption of patient consent to access their personal information for research purposes.

How will participants be recruited into the study? Please provide a detailed step by step description of the recruitment methods i.e. flyers, adverts, direct approach, invitation letter etc.

How will they be approached? Which staff / research team members are approaching the participants? When is this occurring? I.e. clinic, inpatient.

SALHN and Canberra Health Services:

(1) **Participant identification:**

a. A recruitment flyer (Appendix A) will be put on various care settings described above where clinicians will help the researchers identify potential participants. Clinicians (or a site-specific researcher at Canberra Health Services) will identify potential participants based on the inclusion and exclusion criteria, distribute the flyer to them and ask if potential carer participants would like to be contacted by a site-specific researcher. After they have gained carers' permission, researcher, Ying Yu, (or a site-specific researcher at Canberra Health Services) will talk to potential carer about the study and undertake eligibility check.

b. We will also publish recruitment flyer on social media such as Facebook and newsletters via the recruitment sites.

c. Database will be screened by clinicians (or a site-specific researcher at Canberra Health Services) to identify potential participants in various care settings, for

example the memory and geriatric clinics, aged care and dementia care wards, ambulatory geriatric services and falls clinic. Due to the covid restriction, potential participants may not be able to come to clinics for face-to-face follow-up. Therefore, we will use various methods (i.e., posting or emailing the Flyer or talk to them by phone) to gain their expression of interest in the study. They decide whether they would like to participate in the study.

(2) **Eligibility check**: The researcher will check with carer if their care recipient would be interested and available to be assessed. If not, the researcher will undertake cognitive assessment for the care recipients based on carers observation using Global Deterioration Stage between normal ageing and Alzheimer's disease

(3) **Capacity to Consent:** The researcher will check with carer if their care recipient would be interested in and available to be assessed. If yes, the researcher will also use the 'Evaluation to Sign Consent' (Appendix B) (Resnick et al., 2007) to assess the capacity of the care recipients to provide consent for MBS and PBS data used in the study. It will take only 5 minutes for the researcher to complete the assessment. If answer is no, there is no need to do this assessment.

(4) **Explanation**: For potential carer participants who have met the inclusion criteria, the researcher Ying Yu (or a site-specific researcher at Canberra Health Services) will provide them with Information Pack including Participant Information Sheet and Consent Form (PICF) (Appendix C). The researcher will also provide the care recipients with simplified PICF (Appendix D). The researcher will explain details of the research project (including their rights to withdraw) and answer questions.

(5) **Informed consent:** The researcher will contact potential carer participants and their care recipients (only if they want to be involved) at least 24 hours (a maximum of 3 days) to confirm their participation and will meet them face-to-face or online to sign a written consent including the consent for MBS and PBS data used in the study. For the care recipients, a verbal consent will be recorded using voice recorder with their informal carer presents. This is to ensure the conversation can be replayed to the care recipients if cognitive condition declines at the later dates.

(6) **Third-party consent:** If the care recipients have no capacity to make decision to sign consent as indicated by the assessment using the 'Evaluation to Sign Consent', the carers will be requested to be the third-party to sign the consent form (See Appendix D) for sharing information about the care recipients' health resources use using 'The resource utilization for dementia care (RUD) questionnaire' (see Appendix G). In addition, the researcher will also request the carers (or other persons when appropriate), who have a written enduring power of

attorney or legal guardianship documents, to be the third-party to sign the consent form for the person with dementia to allow Services Australia to release MBS/PBS information used in the study (See Appendix E). If the care recipient is not interested in the study, we will not collect information related to the care recipient's 'The resource utilization for dementia care (RUD) questionnaire' (see Appendix G) and MBS/PBS.

Bolton Clarke Victoria and Resthaven SA:

(1) **Participant identification:** In community aged care managed by Bolton Clarke Victoria and Resthaven SA, staff will identify potential participants by checking the database of clients, distribute a recruitment flyer to potential carer participants and ask if they would like to be contacted by a site-specific researcher. After they have gained carers' permission, researcher, Ying Yu, (or a site-specific researcher at Bolton Clarke) will talk to potential carer about the study and undertake eligibility check.

(2) **Eligibility check:** same as described above.

(3) **Capacity to Consent:** same as described above.

(4) **Explanation:** same as described above.

(5) Informed consent: same as described above.

(6) **Third-party consent:** same as described above.

Community

(1) Participant identification: Participant will contact researcher (contacts will be provided via flyer) to gain further information. Researcher, Ying Yu, (or a site-specific researcher at Canberra Health service or Bolton Clarke) will talk to potential participants about the study and undertake eligibility check.

(2) Eligibility check: same as described above.

(3) Capacity to Consent: same as described above.

(4) Explanation: same as described above.

(5) Informed consent: same as described above.

(6) Third-party consent: same as described above.

What are the inclusion and exclusion criteria? - Detail the characteristics that clearly describe the study population that are required to be either included or excluded in the research.

The study population is informal carers of community-dwelling people living with dementia (PLWD).

**Inclusion criteria**<br>The carer need to meet these conditions: (1) The carer is aged 18 years or over; (2) The carer provides care support for an adult person at home living with dementia at least twice a week; (3) if a care recipient has no formal dementia diagnosis but meets the following three criteria: a) cognitive impairment using valid tools such as MMSE to measure (sored <24) or other validated tools Global Deterioration Stage between normal ageing and Alzheimer's disease (GDS), or RUDAS) to measure (Independent Hospital Pricing Authority, 2014; Reisberg, Ferris, de Leon, & Crook, 1982; Storey, Rowland, Conforti, & Dickson, 2004); b) self-care decline and c) changed behaviours using the valid 'Blessed Dementia Dependence Score'(Blessed, Tomlinson, & Roth, 1968).<br>**Exclusion criteria**<br>Carers will be excluded if they (1) have health conditions that may significantly impact their ability to participate in the study; (2) involve in other studies, and (3) cannot read English without additional assistance.
**Participant commitment** -What will their participation involve? I.e. study visits, procedures, tests, tissue samples, questionnaires, wearing of any devices.<br>**Arm 1–Usual care group:** Carers will receive the usual carer support provided by Dementia Australia or other publicly funded carer support. They will receive a monthly reminder email that directs them to the Dementia Australia website where they can seek support if they so wish. Carers will undertake an online survey (Appendix F) at three time points: baseline, 6 months and 12 months. They will be notified about each survey by email and an SMS message. Carer can reply 'stop' to indicate the termination of the reminder and withdrawing from the study. Each survey will take about 30 minutes. Carers will also undertake a monthly 10-minute survey to document the previous month health care services utilisation (Appendix G). Therefore, the time commitment for this group of carers would be no more than 9 hours.<br>**Arm 2–Intervention group:** Carers will engage in these activities:<br>(1) Managing transitions: In each study site, carers will be assigned to an iSupport program facilitator and encouraged to request individualised support from the facilitator during transition between care settings (i.e. from hospital to home transitional care) and care types (i.e. receiving ambulatory geriatric services, or rehabilitation or palliative care at home in addition to usual home care package).<br>(2) Managing dementia progression: Carers are also encouraged to contact the facilitator to discuss changes in PLWD (i.e. signs of deterioration, changed

behaviours and complications) and gain advice to access relevant multidisciplinary care services for timely treatment and care. The facilitator will follow the carers until their needs for support have been met.

(3) Psychoeducation: Carers will select at least 20 out of 30 learning units from the that are relevant to them from the online iSupport program or a hardcopy of iSupport handbook to learn over the first 6 months of the trial and revisit or learn new units when they feel it is needed in the second 6 months. The expected time spent on their engagement with the online iSupport learning units will be no more than 12 hours in total. The completion of each unit will be measured through the program design and will be recorded as data for compliance with the intervention. Carers will receive a certificate for each unit they complete, a strategy to motivate them in the program.

(4) Carer support group: In each study site, the facilitator will assign the 23 carers into one of two support groups. The facilitator will conduct a monthly online carer support group meeting lasting no more than 30 minutes. Managing transitions between care settings and types and managing disease progression will be standard meeting agenda so that carers can share their experiences and support each other in these dementia caregiving areas. The meetings will be recorded for carers in the same group to access. The facilitator will also create carer support groups in WhatsApp to encourage carers to talk or send text messages to their peers in the same group to strengthen social support. Facilitator will communicate with participants via phone calls if they have no access to zoom or WhatsApp. The facilitator will analyse group interactions, investigate carers' needs for support and provide feedback to them.

(5) Feedback on services: The facilitators will collect carers' feedback via regular carer support group meetings, and discuss the feedback in their organisation's quality improvement meetings.

(6) Confidentiality: Carers will follow an agreed protocol to maintain confidentiality and privacy in group activities.

(7) Carers will undertake an online survey at three time points: baseline, 6 months, and 12 months. Each survey will take about 30 minutes. Carers will also undertake a monthly 10-minute survey (online or hardcopy) to document the previous month health care services utilisation. They will be notified about each survey by email and an SMS message sent by the researcher. Carer can reply 'stop' to indicate the termination of the reminder and withdrawing from the study. Therefore, the time commitment for this group of carers would be no more than 27 hours.

**Participant follow up** – how are participants monitored during the study?
All carers in this study will undertake an online survey at three time points: baseline, 6 months and 12 months. The survey will enable researchers to monitor the effectiveness and cost-effectiveness of the education intervention for carers. Carers will also undertake a monthly 10-minute survey (online or hardcopy) to document the previous month health care services utilisation. Carers in the intervention group will also participate in monthly online carer group meeting for 12 months. The meetings will enable researchers to monitor how carers in the intervention group share their learning experience with peers and support each other.

Consent

Please refer to the National Statement 2.2 for guidance on consenting participants.

Where possible, informed consent should be sought from individuals to participate in research or to access their data for research purposes.

Consent can provided in writing, implied (i.e. by return of a survey), opt in, opt out or verbally.

If consent cannot be obtained from the participant, a waiver of consent can be applied for which is reviewed and approved by the SAC HREC. The waiver of consent must be justified using the National Statement chapter 2.3.9 and 2.3.10 (a) to (i) in the HREA.

The investigator(s) should: · Determine, according to level of risk to participants, who of the study team is appropriate to lead the participant informed consent process. This should be documented on the "Delegation of Duties" log. (ICH GCP 5.7)

**How you will be obtaining consent and/or what alternatives you will be using**:
The researcher, Ying Yu (or site-specific researchers to be hired) will explain the study to the potential carer participants and their care recipients; assess eligibility for inclusion based on cognitive assessment of the care recipients; assess the capacity of care recipients to take consent for MBS and PBS data used in the study; provide the PICF (simplified PICF for the care recipients) and MBS and PBS participant consent form, explain the forms to them and allow them sufficient time (at least 24 hours and a maximum of 3 days) to talk to their family and friends about their decision to participate in the study. Once the researcher has confirmed the carers and care recipients' decision to participate in the study, the researcher will sign written consent forms in one of the following methods: 1) post the hardcopy with pre-paid envelop; 2) meet them face to face 3) . Sign the electronic consent.
**Are you requesting a waiver of consent?**

☐ **Yes –** please justify why the waiver of consent is appropriate in the HREA

☒ **No**

Which investigators will issue the information sheets and consent forms:

In SALHN and Resthaven SA, the researcher, Ying Yu who does not provide direct care to the person with dementia or their carers and has no relationship with the dementia care service providers, will issue the information sheets and consent forms to the participants.

In Bolton Clarke Victoria, CI Claudia Meyer who is a Research Fellow at Bolton Clarke Research Institute does not provide direct care to the person with dementia or their carers and has no relationship with the dementia care service providers, will issue the information sheets and consent forms to the participants.

In Canberra Health Services, a site-specific researcher (to be hired) who does not provide direct care to the person with dementia or their carers and has no relationship with the dementia care service providers will issue the information sheets and consent forms to the participants.

How much time will participants have to consider participation:

At least 24 hours and maximum 7 days will be given to potential participants to consider participation. They are encouraged to discuss with their family, friends and colleagues regarding participation.

Please specify which investigators will obtain consent from participants:

In SALHN and Resthaven SA, researcher, Ying Yu who does not provide direct care to the person with dementia or their carers and have no relationship with the dementia care service providers, will obtain consent after detailed explanation of the research project and assessment of eligibility for inclusion based on cognitive assessment.

In Bolton Clarke Victoria, CI Claudia Meyer who is a Research Fellow at Bolton Clarke Research Institute does not provide direct care to the person with dementia or their carers and has no relationship with the dementia care service providers, will obtain consent after detailed explanation of the research project and assessment of eligibility for inclusion based on cognitive assessment.

In Canberra Health Services, a site-specific researcher (to be hired) who does not provide direct care to the person with dementia or their carers and have no relationship with the dementia care service providers, will obtain consent after detailed

explanation of the research project and assessment of eligibility for inclusion based on cognitive assessment.

Will there be an opportunity to confirm or renegotiate consent during the research project? – I.e. the capacity of the participant changes or the terms of consent / participation changes.

(1) After the initial written consent, before each survey, a statement will be made to inform carer participants that by submitting the survey, they consent to participate in the study (implied consent).
(2) Carers will be notified about each survey by email and an SMS message.. Carer can reply 'stop' to indicate the termination of the reminder and withdrawing from the study.
(3) In the intervention group, program facilitator will gain verbal consent from participants to participate in each of carer support group meeting.
(4) A withdraw consent form (Appendix H) will be used if participants wish to withdraw during the research when appropriate.

Who will be confirming or renegotiating consent with participants and what process will be undertaken?

(1) **SALHN and Resthaven SA sites**:
The researcher, Ying Yu who obtained consent form from participants will be confirming or renegotiating consent with participants. She will send SMS to participants to remind them of each follow-up survey. She will also remind participants that they have the right to withdraw at any stage of the study. Participants can reply 'stop' to indicate the termination of the reminder and withdrawing from the study.

(2) **Bolton Clarke Victoria:**
CI Claudia Meyer who obtained consent form from participants will be confirming or renegotiating consent with participants. The researcher will send SMS to participants to remind them of each follow-up survey. The researcher will also remind participants that they have the right to withdraw at any stage of the study. Participants can reply 'stop' to indicate the termination of the reminder and withdrawing from the study.

(3) Canberra Health Services
A site-specific researcher (to be hired) who obtained consent form from

participants will be confirming or renegotiating consent with participants. The researcher will send SMS to participants to remind them of each follow-up survey. The researcher will also remind participants that they have the right to withdraw at any stage of the study. Participants can reply 'stop' to indicate the termination of the reminder and withdrawing from the study.

Conflicts of interest: Please refer to the National Statement chapter 5.4, and your institutional policy for guidance.

☒ Yes / ☐ No

Please provide details of the conflict of interest:

Four team members are employed in a management role in participating organisations in the project. Therefore, they have potential conflict of interest. Ms Langduo Chen is the Unit manager for Rehabilitation Ward, Southern Adelaide Local Health Network (SALHN) and Dr Craig Whitehead is the Regional Clinical Director for Rehabilitation and Aged Care, SALHN. Therefore, they have work relationships with people with dementia at SALHN. Dr Michael Chapman is the Director, Palliative Care at Canberra Health Services and has work relationships with people with dementia at Canberra Health Services. Ms Sue McKechnie is the Executive Manager Community Services, Resthaven Incorporated at Resthaven SA. She has a leadership and management role in community aged care where participants will be recruited to the study.

How will the conflict be managed?

The potential conflict of interest will be managed by maintaining the confidentiality of all participants' data. These four members will not participate in the participant recruitment and data collection. Each participant in the study will be given a unique code. Only the PI, Professor Xiao, and site-specific researchers employed to collect data will have the record of the participants' real name and code number. However, the four team members who have potential conflict of interest will be able to access de-identified data when participate in data analysis and in reporting findings.

Ethical considerations

Please describe the risk and burden associated with your research. The National Statement chapter 2.1 provides guidance and advice on the definition of risk and how to gauge and manage it.

As an online psychoeducation intervention for carers of people with dementia and health

service research, we do not foresee any significant risks to any participants of this project. The foreseen risks are time burden on carers, especially those in the intervention group. Further, Carers may experience emotional, psychological distress from recalling experiences during the online survey or carer support group meetings. In addition, confidentiality of information may be breached that will threaten participants' privacy.
**How will any risks be managed?**<br>**Time burden:**<br>These risks will be managed by self-paced learning. Carers will choose the time and date that are suitable for them to engage in the online iSupport program. Participants also have the rights to choose the time that is convenient for them to participate in the online carer support group meetings. Any carer who feels the time commitment is too great to complete a given component of the study is under no compulsion to participate or may withdraw from the study at any point. Carers will receive $50 per month for 12 months per person as a token of appreciation for their time contributing to the study.<br>**Emotional and psychological distress:**<br>In the online survey, participants will be instructed that they have the rights to choose whether they would like to answer some questions or not. In the carer support group meetings, the program facilitator will verbally instruct participants that they have the rights to choose whether they would like to answer some questions or not. They can leave the group discussion at any time if they do not feel comfortable in sharing their experiences. If they experience feelings of distress as a result of participation in this study, they are encouraged to let the research team know immediately. They will be offered counselling service or refer to relevant emergency service if required. They can also contact the following services for support:<br>Dementia Australia – 1800 100 500<br>Lifeline – 13 11 14, www.lifeline.org.au<br>Beyond Blue – 1300 22 4636, www.beyondblue.org.au<br>**Potential breach of confidentiality of information**<br>Program facilitators will verbally remind carers in each carer group meeting to maintain confidentiality of information discussed in the group. The voice recorded meeting will be transcribed by Ying Yu using NVivo Transcription software. Transcripts from group meetings and text messages will be de-identified by the researcher before the data analyses. Regarding the data received from MBS and PBS, the team will need to provide identifiable personal information to Services Australia to ensure data is extracted only for participants who have consented to the release of their data for this trial. However, this will be done via a password protected and encrypted file sent directly via registered post to Services

Australia, as per their requirements. The data from MBS and PBS will be sent back directly to the researcher conducting the analysis at Flinders University (Dr Rachel Milte) and will be in de-identified format (i.e. all personal details removed). This information will be kept securely and separately from any data with identifying information containing identifying data to reduce the risk of any breaches in privacy and confidentiality.
**Benefits –** please identify and explain the expected outcomes and benefits of the study<br><br>This study aligns to the National priority areas in the Dementia, Ageing and Aged Care Mission Roadmap as it creates an innovative patient-centred care approach built on reciprocal partnerships between care service providers and carers that will reduce carers' stress and enable carers to manage dementia well at home and to prevent health conditions contributing to hospitalisations of PLWD. Therefore, these outcomes will improve health and QoL for PLWD and their carers and will ease the impact of dementia on the health care system. Further, the study will strengthen proactive health management by providing carers with psychoeducation which improves their ability to manage dementia and prevent changed behaviours and complications.<br><br>In addition, the 'Partnership in iSupport program' is an innovative patient-centred care model that targets the organisation's structures, resources and processes to improve care services. The program will be implemented by four industry partners in the project. Findings will provide evidence on the efficacy of an online education program in dementia care and in virtual social support that meet carers' needs. This program is designed to help carers seek practical solutions to their individual issues from the service provider and peers in a timely manner to prevent care crises that may trigger hospital admission, emergency department use and premature admission to residential aged care homes. The program is also designed to improve reach to carers in rural and remote areas and to carers of working age. The COVID-19 pandemic has highlighted the urgent need to have programs such as iSupport which are not impacted by lockdowns.
**Does a dependant or unequal relationship exist between the participant and the researcher?**<br><br>Please refer to the National Statement 4.3 for advice and guidance on how to manage this**.**<br><br>☐ Yes -<br><br>How will the dependant / unequal relationship be managed? Click here to enter text.<br><br>☒ No

Data management – as required in addition to that outlined in your HREA

As per the National Statement 3.1.45, researchers must have a data management plan in place.

The Office for Research would like to remind researchers that the disposal of research records must be made in accordance with The State Records Act 1997 (the Act). Under that Act records must be disposed of as outlined in the general disposal schedules.

Public health institutions fall under general disposal schedule 28. As per item 6 of general disposal schedule 28, the researchers records of research including results, notes, completed questionnaires, signed consent forms, data, reports, and study findings must be kept for 15 years after the research project has been completed before being destroyed. This includes all types of research.

Universities fall under general disposal schedule 24. As per section 9 of general disposal schedule 24 research data records should be kept for a duration according to the nature of the study. For short term research projects such as study research projects, data should be kept for 1 year after last action. Research data from clinical trials should be kept for 15 years after action completed. All other research data and results should be kept for 5 years after publication, conclusion, or abandonment of the project. Data should be destroyed after the mandatory retention period.

Unless informed consent has been obtained from the participant, or legally authorised person, or the HREC has expressly approved otherwise, personal information used or disclosed for research purposes, must be de-identified. Only SA Health employees will perform the de-identification process prior to releasing the information for research purposes.

Who will collect the study data / information? Only SA Health employees can access patient data for research purposes. Students and non-SA Health employees cannot access patient records for research purposes under any circumstances.

The researchers will not access participants' data from hospitals for research purposes.

Baseline demographic data and care service utilisation data:

SALHN and Resthaven SA: The Flinders University employed researcher, Ying Yu, will collect baseline demographic data and a monthly care service utilisation data using RUD using the secured Flinders University online survey platform.

Bolton Clarke Victoria: Dr Claudia Meyer at the Bolton Clarke Research Institute will collect baseline demographic data and care service utilisation data.

Canberra Health Services: A researcher (name to be provided) employed by CI Dr Michael Chapman will collect baseline demographic data and care service utilisation data.

Self-administered survey:

In addition to above data collection, participants will participate in online self-administered survey three times at baseline, 6 months and 12 months as part of data collection methods for the analyses of program effectiveness.<br>**Online care support group meeting data:**<br>The monthly online care support group meetings in the intervention group will be audio-recorded for data analysis to understand carers' experience in the iSupport program.<br>**Messages between participants and program facilitator:**<br>The messages (text messages via mobile, email and WhatsApp, request for support) and time spent by facilitator to provide the support will be documented and analysed.<br>**MBS and PBS data:** Will be provided by Services Australia, subject to their own approval process and requirements.
**What format will the data or information be stored**?<br>**Digital audio data format:** Data collected in the online carer group meetings, recorded/downloaded text messages and the voice recording of verbal consent are digital audio data. These types of data will be stored in a digital audio data format.<br>**Data in hard copy format**: The expression of interest and signed consent forms will be hardcopy data.<br>**Electronic data format**: The digital audio data from carer group meetings will be transcribed verbatim and the transcripts will be checked by researchers in each study site. Participants' identities will be removed from the transcripts before the data analysis. The de-identified transcripts will be stored in electronic format.<br>Online survey data without participant identities will be stored in an electronic data format.<br>Any data received from Services Australia will be stored in a password protected electronic folder on the Flinders University Research Drive. Only the PI Prof Lily Xiao and the health economists Prof Julie Ratcliffe and Dr Rachel Milte can access the folder to conduct the analysis or maintain proper governance and maintenance of the data files.
**Please provide details regarding training of the research team on maintaining the integrity and security of the data –**<br>The research team have relevant experience in maintaining the integrity and security of the type of data we will collect for this study. Prior to the study, all program facilitators researchers will attend an online training session to standardise data

collection and data analysis, and to comply with the study protocol and ethics requirements. PI Professor Xiao will lead the training session.

Strategies as listed below will be applied to maintain the integrity and security of the data:

(1) The data will be verified and validated by two researchers to ensure the input is accurate.
(2) The research team will secure the data with limited system access. For example, during the data collection and data analysis period, raw data will be in electronic format, have the unique codes for participants and be kept in password-protected electronic file. Only researchers in the project can access the data.
(3) Ms Ying Yu will undertake monthly audits across study sites regarding the compliance with confidentiality of information and data storage.

What conditions can the data be accessed or granted to others?

De-identified online survey data in SPSS format, the transcripts of the online carer support group meetings and text messages will be shared amongst the research team only. No identifying information will be shared. No data received from Services Australia will be used for purposes outside this research project or provided to anyone outside of the research team.

How will the research data be stored and what security measures are in place to protect it?

Digital audio data:

The digital audio data collected from all study sites will be submitted to the PI Xiao and will be stored securely on the Research Drive at Flinders University by the PI Xiao. The data will not be shared within the project team, but for audit purpose.

Electronic data format:

The de-identified transcripts of online carer support group meetings and downloaded deidentified text messages will be stored in a password protected folder on the Flinders University Research Drive. This folder will be shared with all researchers in the project during this study phase for data analysis purpose. Once the data analysis has been completed, only the PI Prof Lily Xiao can access the folder to maintain proper governance.

The online survey data will be downloaded and stored in an electronic data format in a password protected folder on the Flinders University Research Drive. Only the PI Xiao, CI Ullah (biostatistician) and the researcher Ying Yu can access the folder to conduct

the analysis. On the completion of this phase of study, only the PI Xiao can access the folder to maintain proper governance. The site-specific CIs will scan the expression of interest and signed consent forms and store the scanned forms in an electronic data format in a password protected folder on the Flinders University Research Drive. Text messages will be downloaded as a On the completion of the data submission, only the PI Prof Lily Xiao can access the folder to maintain proper governance.<br>The originals hard copy of the MBS and PBS consent form MUST be provided to Services Australia. The research Dr Milte will scan the signed consent forms and store the scanned forms in an electronic data format in a password protected folder on the Flinders University Research Drive. Only the PI Xiao can access the folder to maintain proper governance<br>Any data received from Services Australia will be stored in a password protected electronic folder, on a password protected Flinders University Research Driver. Only the PI Prof Lily Xiao, CI Ratcliffe and Dr Rachel Milte can access the folder to conduct the analysis during this study phase. On the completion of this phase of study, only the PI Xiao can access the folder to maintain proper governance or maintain proper governance and maintenance of the data files.<br>**Data in hardcopy format:**<br>The hard copy data collected by each study site will be stored in a secure locked filing cabinet in a particular room in a particular building approved by the organisation of the study site. Only the lead CI in each study site (PI Xiao at Flinders University, CI Meyer at Bolton Clarke Research Institute and CI Chapman at Canberra Health Services) can access the data to maintain proper governance.
**How will you provide access to, disclose, use/re-use or transfer the data**?<br>Data will be analysed collectively in the project team. Therefore, de-identified data stored on the secured cloud of Flinders University Research Drive, will be used to access and share the data within the team members across multiple study sites during the study phase. To protect participants' identity, all sensitive (audio-recordings) and demographic information will be stored on the Research Drive and no access will be given to the broader research team. Participants will be assured that pseudonyms will be used in the reports and publications of the project.
**How long will the data be retained for?**<br>☒ The data will be kept for 15 years – for all SA Health research<br>☐ The data will be kept for 5 years – for all University research,

**What plans are in place to store / archive the study data once the research is completed**?<br>The Chief Investigator, Professor Xiao, will maintain all records pertaining to this study for 15 years after publication according to the Australian Code for Responsible Conduct in Research. After 15 years, records will be destroyed according to the Government of South Australia General Disposal Schedule No. 24/30.<br>**What is the archive plan if the chief investigator leaves the institution and no longer has access to the study data?**<br>On the completion of the study, data will be stored on the Flinders University Research Drive which can be owner transferred to the associate investigator of the team by IT staff if the Chief Investigator, Professor Xiao, leaves the institution and no longer has access to the study data.
**How will the study data be destroyed**?<br>All data will be retained for 15 years, and then it will be shredded to destroy permanently. Data stored electronically will be deleted from the Flinders University Research Drive and computer permanently by Flinders University IT services.
**Matching and sampling strategies**:<br>**Matching:**<br>Any information provided by participants will be collected in a de-identifiable form. Each participant and study site will be given a unique numerical code to represent their names on the data collection form throughout the project. The code will be used to match data generated from three-time surveys in the study.<br>**Sampling strategies**<br>After baseline data collection, carers will be randomly assigned to receive either the iSupport program group or the usual care group. To ensure the two groups are of equivalent size, a randomly generated block size of 4 will be used to allocate carers to one of the two treatment groups for each recruitment site. The randomisation will ensure equivalent distribution of spouse carers versus non-spouse carers and care recipients with mild versus moderate dementia in each treatment group.
**Accounting for potential bias, confounding factors and missing information**:<br>Dr Shahid Ullah who does not involve in participants recruitment will randomly assign participants (deidentified) into the intervention group and usual care group. This approach will account for potential bias.

Confounding factors will be controlled through statistical analysis design. In order to control the interference of the confounding factors on the research results and reduce the potential bias, univariate models will be first used, then multivariate modelling will be undertaken by adding variables considered clinically important or statistically significant from the univariate model to adjust for confounding effects between variables. A series of models will be undertaken by adding and subtracting variables, with changes in model fit assessed by log likelihood to choose the final multivariate model.<br><br>Data will be analysed on an intention-to-treat basis based on group assignments. For missing information in the data analysis, Mean/Model Imputation will be used to reduce the bias in missing data completely at random.
**Sample size and statistical or power issues** – Make sure the size and profile of the sample to be recruited is adequate to answer the research question – please provide details:<br>The sample size calculation is based on the primary outcome of the SF-12 mental component for carers and is estimated on the basis of an earlier RCT with similar intervention components for carers (Berwig et al., 2017). In that study, the SF-12 mental component for carers increased significantly by an effect size of 0.57 and standard deviation of 8.63. We assume that the same effect size would be observed in our trial. The estimated sample size would be 66 carers per arm using a 2-sample comparison of means for alpha=0.05 at 90% of power. The Welch-Satterthwaite t-test is used to calculate the sample size for unequal variances. Assuming an attrition rate of 40%, the estimated sample size would be 184 carers in total or 92 per group. A stata code power was used to calculate the sample size. Our investigation indicates that each industry partner provides care services for more than 500 persons with dementia annually and more than 80% of carers use email to communicate with care staff which will ensure sufficient numbers of carers recruited into the study.
**How will you measure, manipulate and/or analyse the information collected**?<br><br>**Measuring the effectiveness of the iSupport program:**<br>A research assistant, who will be blinded to group assignments, will undertake data analysis and will be supervised by CI Ullah, a biostatistician. Data will be analysed on an intention-to-treat basis based on group assignments. Descriptive statistics will be applied to summarise data. Binary correlation analysis will be performed to compare group using baseline data. A multivariate mixed effect linear regression model will be

applied to fit linear mixed models to examine the primary and secondary outcomes between groups. As the outcome occurs for each individual with repeated time points, the mixed effect models will capture both fixed effects and random effects within the hierarchical structure of the data. The fixed effects, including group effect, time effect and group x time interaction, will be analogous to the regression coefficients. The random effects represent the estimated variability in the intercept to account for repeated measurements. The model will be adjusted by the baseline measure of outcome variable. The maximum likelihood estimate procedure will be used to compare significant differences in primary and secondary outcomes over time and between groups. Univariate models will be first used, then multivariate modelling will be undertaken by adding variables considered clinically important or statistically significant from the univariate model to adjust for confounding effects between variables. A series of models will be undertaken by adding and subtracting variables, with changes in model fit assessed by log likelihood to choose the final multivariate model. The two-sided test will be performed for all analyses and the level of significance will be set at $p < 0.05$. All analyses will be performed using Stata software version 16.1.

Exploring carers' experience in the iSupport program:
PhD student, Ying Yu will analyse the qualitative data collected from online carer support groups meetings and downloaded text messages between participants and facilitators. She will be supervised by PI Professor Xiao in data analyses. Audio-recorded meetings data will be transcribed verbatim for analysis using NVivo Transcription software. Transcripts will be entered into a computer-assisted qualitative data analysis program, NVivo13, for data management to facilitate coding. Thematic analysis through coding, grouping codes and summarising codes into themes will be applied (Nowell, Norris, White, & Moules, 2017). Findings will be discussed in regular team meetings to reach consensus.

Measuring the cost-effectiveness of the iSupport program:
The cost effectiveness analysis will be undertaken by Dr Rachel Milte and will be supervised by CI Professor Julie Ratcliffe. The primary outcome will be the incremental cost per quality adjusted life years (QALYs) gained for the intervention compared to usual care. Resources associated with the development and implementation of the intervention will be documented and costed according to established best practice guidelines (Drummond, Sculpher, Claxton, Stoddart, & Torrance, 2015). The primary measure of effectiveness will be the incremental gains in

QALYs from the perspective of carers and people with dementia. SF-12 responses between baseline and 6 months will be converted into health state utilities for the calculation of QALYs for carers using the SF-6D preference based scoring algorithm developed by Brazier and colleagues (Brazier et al., 2017). DEMQOL-Proxy responses will be similarly converted using the preference-based scoring algorithm developed by Mulhern and colleagues (Mulhern et al., 2013). Data on health and social care service use for carers and care recipients will be collected via interview from the carers using the RUD monthly. Pre-baseline resource use data will provide additional baseline variables to control for potential confounding. The difference between pre- and post-baseline use of health services will be used to measure changes in carers' and care recipients' levels of utilisation and cost of health services for participants in both study arms.<br><br>Unit costs will be derived from following guidelines for conducting economic evaluations in Australia from published sources provided by Medicare, hospital finance departments and Australian Refined Diagnosis Related Groups cost weights. Cost of nursing home care will be calculated using basic daily and accommodation fees apportioned according to length of stay. An assessment of the sensitivity of the results obtained to variation in measured resources, effectiveness and/or unit costs will be undertaken using appropriate one-way and multi-way sensitivity analysis. A budget impact analysis will also be conducted, extrapolating the within trial assessment of costs and outcomes to a population level.
**Data linkage** –what linkages are planned or anticipated?<br>Medicare Benefits Schedule (MBS) and/or Pharmaceutical Benefits Scheme (PBS) data of carers and care recipients will be extracted to collect information on health services utilisation. MBS and PBS consent form provided by Services Australia (see Appendix E) will be used with the participants to gain consent to allow Services Australia to release MBS and PBS data. The data provided by Services Australia will be linked to other data collected by online survey, self-report with the participants (for example information responses to surveys about their health and quality of life and basic demographic information (for example age, gender) and caregiver status as well as use of other health and social care resources collected via the RUD questionnaire. This will enable the cost-effectiveness analysis for the project to occur.
**What impact will a participant withdrawing have on the data and how will this be responded to**?

We have assumed an attrition rate of 40% in sample size calculation. This sample size will ensure a participant withdrawing has little impact on the data and statistical power. A clear flow form about the numbers of participants' recruitment, allocation, withdraw, and analysis will be reported in the project reports and publications to foster transparency of the study.<br>A withdraw consent form will be used if participants wish to withdraw during the research. Participants will be informed that data collected up to the time of withdraw will be used unless they suggest otherwise.

Results, reporting, outcomes and future plans

Post approval monitoring and reporting

Once you have received ethics and governance authorisation for your research project, there are mandatory reporting requirements you must adhere to as per The National Statement chapter 5.5.

- Annual review – this is required annually for the life of the audit, on the anniversary of the approval date. Please use the template on our website.
- Final report – this is required to be submitted on completion of the audit. Please use the template on our website

Please refer to the Office for Research Reporting and Monitoring guidelines on our website for the mandatory reporting requirements for this research project.

Failure to submit the required reports of a breach of the NHRMC Australian Code for the Responsible Conduct of Research R17, R22, the National Statement chapter 5.5 and the terms and conditions of the ethical approval of the study. Failure to submit the required report may result in the ethics approval being withdrawn and the application closed.

**Please detail your plans for the return of the research results to the participants**:<br>The researchers will provide the participating organisations with a written summary of findings from the study. The summary will be ready for participating organisations to use in their newsletters and website. Therefore, participants in the project will be informed of the main findings of the project.
**What are your plans for dissemination and publication of project outcomes**:<br>The outcome will be published to journals. The result will be shared and disseminated through conference and inhouse education.
**Please detail other potential uses of the data at the end of the project**:<br>N/A
**What are your plans for sharing and/or future use of data and/or follow-up research?** i.e. anticipated secondary use of data:

N/A
**What is the project closure process?** I.e. a final report will be submitted to the HREC, where the study data and/or samples will be stored?<br>1. The final report will be produced and submitted to the HREC, MRFF/Dementia Collaborative and Research Centre.<br>2. Finding of the study will be reported to participants via email when the research project is completed.<br>3. The outcomes in this project will be published in the publications related to this topic.

Reference

Alzheimer's Australia. (2015). *Caring for someone with dementia: The economic, social and health impacts of caring and evidence-based support for carers*. Retrieved from

Alzheimer Scotland. (2019). Scotland's National Dementia Strategy (2017 - 2020).

Anderson, T. S., Marcantonio, E. R., McCarthy, E. P., & Herzig, S. J. (2020). National Trends in Potentially Preventable Hospitalizations of Older Adults with Dementia. *Journal of the American Geriatrics Society, 68*(10), 2240-2248. doi:https://doi.org/10.1111/jgs.16636

Berwig, M., Heinrich, S., Spahlholz, J., Hallensleben, N., Brahler, E., & Gertz, H. J. (2017). Individualized support for informal caregivers of people with dementia-effectiveness of the German adaptation of REACH II. *Bmc Geriatrics, 17*(286). doi:10.1186/s12877-017-0678-y

Blessed, G., Tomlinson, B., & Roth, M. (1968). "The association between quantitative measures of dementia and of senile change in the cerebral grey matter of elderly subjects. *British Journal of Psychiatry, 114*(512), 797-811.

Bott, N. T., Sheckter, C. C., Yang, D., Peters, S., Brady, B., Plowman, S., . . . Milstein, A. (2019). Systems delivery innovation for Alzheimer disease. *American Journal of Geriatric Psychiatry, 27*(2), 149-161. doi:10.1016/j.jagp.2018.09.015

Brazier, J., Ratcliffe, J., Salomon J, T., & Tsuchiya, A. (2017). *Measuring and valuing health benefits for economic evaluation* (2nd ed.). Oxford, UK: Oxford University Press.

Bressan, V., Visintini, C., & Palese, A. (2020). What do family caregivers of people with dementia need? *Health & Social Care in the Community, 28*(6), 1942-1960. doi:10.1111/hsc.13048

Cepoiu-Martin, M., Tam-Tham, H., Patten, S., Maxwell, C. J., & Hogan, D. B. (2016). Predictors of long-term care placement in persons with dementia. *International Journal of Geriatric Psychiatry, 31*(11), 1151-1171. doi:doi:10.1002/gps.4449

Cheng, S. T., Li, K. K., Losada, A., Zhang, F., Au, A., Thompson, L. W., & Gallagher-Thompson, D. (2020). The Effectiveness of Nonpharmacological Interventions for Informal Dementia Caregivers. *Psychology and Aging, 35*(1), 55-77. doi:10.1037/pag0000401

Comans, T. A., Nguyen, K.-H., Ratcliffe, J., Rowen, D., & Mulhern, B. (2020). Valuing the AD-5D Dementia Utility Instrument. *PharmacoEconomics, 38*(8), 871-881. doi:10.1007/s40273-020-00913-7

Denzin, N. (2017). *The research act : a theoretical introduction to sociological methods*. London, England, New York, New York: Routledge.

Doyle, L., McCabe, C., Keogh, B., Brady, A., & McCann, M. (2019). An overview of the qualitative descriptive design within nursing research. *Journal of Research in Nursing, 25*(5), 443-455. doi:10.1177/1744987119880234

Drummond, M., Claxton, K., Sculpher, M., O'Brien, B., Stoddart, G., & Torrance, G. (2015). *Methods for the Economic Evaluation of Health Care Programmes* (4th ed.). Oxford, UK: Oxford University Press.

Drummond, M. F., Sculpher, M. J., Claxton, K., Stoddart, G. L., & Torrance, G. W. (2015). *Methods for the Economic Evaluation of Health Care Programmes.* (4th ed. ed.): Oxford, UK: Oxford University Press.

Dyer, S., van den Berg, M., Barnett, K., Brown, A., Johnstone, G., Laver, K., . . . Crotty, M. (2019). *Review of Innovative Models of Aged Care*. Retrieved from Adelaide, Australia.:

Egan, K. J., Pinto-Bruno, A. C., Bighelli, I., Berg-Weger, M., van Straten, A., Albanese, E., & Pot, A. M. (2018). Online Training and Support Programs Designed to Improve Mental Health and Reduce Burden Among Caregivers of People With Dementia: A Systematic Review. *Journal of the American Medical Directors Association, 19*(3), 200-U142. doi:10.1016/j.jamda.2017.10.023

Etxeberria, I., Salaberria, K., & Gorostiaga, A. Online support for family caregivers of people with dementia: a systematic review and meta-analysis of RCTs and quasi-experimental studies. *Aging & Mental Health*. doi:10.1080/13607863.2020.1758900

Frias, C. E., Garcia-Pascual, M., Montoro, M., Ribas, N., Risco, E., & Zabalegui, A. (2020). Effectiveness of a psychoeducational intervention for caregivers of People With Dementia. *Journal of Advanced Nursing, 76*(3), 787-802. doi:10.1111/jan.14286

Goeman, D., Renehan, E., & Koch, S. (2016). What is the effectiveness of the support worker role for people with dementia and their carers? *BMC Health Serv Res, 16*(1), 285-285. doi:10.1186/s12913-016-1531-2

Greenwood, N., Mezey, G., & Smith, R. (2018). Social exclusion in adult informal carers. *Maturitas, 112*, 39-45. doi:10.1016/j.maturitas.2018.03.011

Independent Hospital Pricing Authority. (2014). *Standardised Mini-Mental State Examination (SMMSE): Guidelines for administration and scoring instructions*. Retrieved from https://www.ihpa.gov.au/sites/default/files/publications/smmse-guidelines-v2.pdf

Logsdon, R. G., Gibbons, L. E., McCurry, S. M., & Teri, L. (2002). Assessing quality of life in older adults with cognitive impairment. *Psychosom Med, 64*(3), 510-519. doi:10.1097/00006842-200205000-00016

Moholt, J.-M., Friborg, O., Skaalvik, M. W., & Henriksen, N. (2018). Psychometric validation of the Carers of Older People in Europe Index among family caregivers of older persons with dementia. *SAGE Open Medicine, 6*. doi:10.1177/2050312118792812

Mulhern, B., Rowen, D., Brazier, J., Smith, S., Romeo, R., Tait, R., . . . Banerjee, S. (2013). Development of DEMQOL-U and DEMQOL-PROXY-U: generation of preference-based indices from DEMQOL and DEMQOL-PROXY for use in economic evaluation. *Health Technology Assessment, 17*(5), v-140.

Nowell, L. S., Norris, J. M., White, D. E., & Moules, N. J. (2017). Thematic Analysis: Striving to Meet the Trustworthiness Criteria. *International Journal of Qualitative Methods, 16*(1). doi:10.1177/1609406917733847

Oliver, D. P., Patil, S., Benson, J. J., Gage, A., Washington, K., Kruse, R. L., & Demiris, G. (2017). The Effect of Internet Group Support for Caregivers on Social Support, Self-Efficacy, and Caregiver Burden: A Meta-Analysis. *Telemedicine and E-Health, 23*(8), 621-629. doi:10.1089/tmj.2016.0183

Ratcliffe, J., Chen, G., Khadka, J., Kumaran, S., Hutchinson, C., Milte, R., . . . Batchelor, F. (2020). *Australia's aged care system*. Retrieved from South Australia:

Reisberg, B., Ferris, S., de Leon, M., & Crook, T. (1982). The Global Deterioration Scale for assessment of primary degenerative dementia. . *Am J Psychiatry, 139*, 1136-1139.

Resnick, B., Gruber-Baldini, A. L., Pretzer-Aboff, I., Galik, E., Buie, V. C., Russ, K., & Zimmerman, S. (2007). Reliability and Validity of the Evaluation to Sign Consent Measure. *The Gerontologist, 47*(1), 69-77. doi:10.1093/geront/47.1.69

Stall, N. M., Kim, S. J., Hardacre, K. A., Shah, P. S., Straus, S. E., Bronskill, S. E., . . . Rochon, P. A. (2019). Association of Informal Caregiver Distress with Health Outcomes of Community-Dwelling Dementia Care Recipients. *Journal of the American Geriatrics Society, 67*(3), 609-617. doi:10.1111/jgs.15690

Steffen, A. M., McKibbin, C., Zeiss, A. M., Gallagher-Thompson, D., & Bandura, A. (2002). The Revised Scale for Caregiving Self-Efficacy. *The Journals of Gerontology: Series B, 57*(1), P74-P86. doi:10.1093/geronb/57.1.P74

Steiner, G. Z., Ee, C., Dubois, S., MacMillan, F., George, E. S., McBride, K. A., . . . Hohenberg, M. I. (2020). "We need a one-stop-shop". *BMC Geriatrics, 20*(1), 49. doi:10.1186/s12877-019-1410-x

Storey, J. E., Rowland, J. T. J., Conforti, D. A., & Dickson, H. G. (2004). The Rowland Universal Dementia Assessment Scale (RUDAS): a multicultural cognitive assessment scale. *International Psychogeriatrics, 16*(1), 13-31. doi:10.1017/s1041610204000043

Teri, L., Truax, P., Logsdon, R., Uomoto, J., Zarit, S., & Vitaliano, P. P. (1992). Assessment of behavioral problems in dementia. *Psychology and Aging, 7*(4), 622-631. doi:10.1037/0882-7974.7.4.622

Ware, J., Jr., Kosinski, M., & Keller, S. D. (1996). A 12-Item Short-Form Health Survey. *Medical Care, 34*(3), 220-233.

Wimo, A., Wetterholm, A., Mastey, V., & Winblad, B. (1998). Evaluation of the resource utilization and caregiver time in Anti-dementia drug trials - a quantitative battery. In A. Wimo, G. Karlsson, B. Jönsson, & B. Winblad (Eds.), *The Health Economics of dementia*. London, UK: Wiley´s.

Xiao, L. (2020). Adapting iSupport for Australian carers. *Australian Journal of Dementia Care, 8*(6), 12-13.

Xiao, L., McKechnie, S., Jeffers, L., De Bellis, A., Beattie, E., Low, L.-F., . . . Pot, A. M. (2020). Stakeholders' perspectives on adapting the World Health Organization iSupport for Dementia in Australia. *Dementia: the international journal of social research and practice, in press*.
