# Supplementary material for: Effects of a virtual iSupport Program on carers and people with dementia
Source: Alzheimers Dement. 2025 Sep 29;21(10):e70747. doi: 10.1002/alz.70747 (PMC12479211; doi:10.1002/alz.70747)
Supplement: Supplementary file 5 — Supporting Information [file ALZ-21-e70747-s002.docx]

**Supplementary file 4: The training and support for iSupport facilitators**

| Learning sessions | Learning content or activities | Hours |
| --- | --- | --- |
| Completion of the online iSupport program with an achievement of 100% correct answers to exercises. | Self-learning using the online Chinese iSupport program. | 10-15 hours |
| Introduction to the virtual Partnership in iSupport Program. | Online session with Q&A delivered by the project leader. | One hour session. |
| iSupport facilitators’ roles and responsibilities in hospital and community care settings. | Online interactive session delivered by four site-leaders. | One hour session. |
| Communication and information technology (CIT) used in the program | Online interactive session delivered by project team member specialised in CIT and the project coordinator. | Two 1-hour sessions. |
| Health economic study in the program. | Online interactive session delivered by a project team specialised in the health economic study. | One hour session. |
| Self-directed learning: 1) Australian dementia care policies and practices; 2) Case scenarios-based guidance for iSupport facilitators. | Recorded session from two Associated Investigators in the project. | Five hours. |
| Ongoing support for facilitators | Online debrief sessions led by the project leader. | Bi-weekly 30-minute sessions over the 12-months intervention. |
