# Supplementary material for: Effects of a virtual iSupport Program on carers and people with dementia
Source: Alzheimers Dement. 2025 Sep 29;21(10):e70747. doi: 10.1002/alz.70747 (PMC12479211; doi:10.1002/alz.70747)
Supplement: Supplementary file 6 — Supporting Information [file ALZ-21-e70747-s001.docx]

**Supplementary file 5: A meeting agenda for peer support meetings**

| 1. The facilitator welcomes carers to the meeting |
| --- |
| 1. The facilitator explains the ground rules which emphasise equal opportunity for participation, respecting others and maintaining confidentiality for what carers shared in the meeting. |
| 1. The facilitator moderates the discussion to allow each carer to share their experiences in the program and clarify issues when needed. |
| 1. The facilitator prompts carers to share their experiences in the iSupport program and uses these probe questions when appropriate: |
| - 1. What have you studied from the iSupport manual? |
| - 1. How have the iSupport program changed your thoughts in dementia care? |
| - 1. How have the iSupport program changed your interactions with your care recipients? |
| - 1. What difficulties have you experienced as a carer? |
| - 1. What positive experiences have you experienced as a carer? |
| 1. The facilitator briefly summarises the discussion, encourages carers to engage in the program and closes the meeting. |
