# Supplementary material for: Effects of a virtual iSupport Program on carers and people with dementia
Source: Alzheimers Dement. 2025 Sep 29;21(10):e70747. doi: 10.1002/alz.70747 (PMC12479211; doi:10.1002/alz.70747)
Supplement: Supplementary file 7 — Supporting Information [file ALZ-21-e70747-s004.docx]

**Supplementary file 6 Table S1: Changes in outcomes at 6 months and 12 months**

| Outcome measures | Gro-ups | Base-line | 6 M | 12 M | Within-group effect | | Between-group effect | | | |
| --- | --- | --- | --- | --- | --- | --- | --- | --- | --- | --- |
|  |  |  |  |  | 6 M vs. baseline (95%CI) | 12 M vs. baseline (95%CI) | Differences at 6 M (95%CI) | p | Differences at 12 M (95%CI) | p |
| Carer QoL: MCS * | IG | 38.72 | 45.65 | 47.22 | 7.26 (3.77, 10.74)^✝^ | 9.35 (6.00, 12.70)^✝^ | 7.48 (3.29, 11.66) | **<0.001** | 11.99 (7.81, 16.18) | **<0.001** |
|  | UCG | 41.50 | 41.01 | 39.18 | -0.22 (-4.23, 3.79) | -2.64 (-6.16, 0.87) |  |  |  |  |
| Carer QoL: PCS * | IG | 46.14 | 48.17 | 42.78 | 1.45 (-2.65, 5.55) | -4.72 (-9.21, -0.24)^✝^ | 3.23 (-1.48, 7.95) | 0.179 | 3.96 (-0.76, 8.67) | 0.100 |
|  | UCG | 48.10 | 46.23 | 40.76 | -1.78 (-5.56, 2.00) | -8.68 (-12.12 -5.25)^✝^ |  |  |  |  |
| CSE: respite * | IG | 45.36 | 46.58 | 53.16 | 1.39 (-6.80, 9.57) | 8.79 (1.26 16.32)^✝^ | 0.32 (-8.68, 9.32) | 0.944 | 14.80 (5.79, 23.80) | **0.001** |
|  | UCG | 46.53 | 48.61 | 40.85 | 1.07 (-7.46, 9.60) | -6.01 (-14.34, 2.33) |  |  |  |  |
| CSE: behaviours * | IG | 70.97 | 77.51 | 82.50 | 6.53 (1.86, 11.19) ^✝^ | 11.15 (6.32, 15.98)^✝^ | 6.16 (0.23, 12.08) | **0.042** | 15.56 (9.64, 21.48) | **<0.001** |
|  | UCG | 70.26 | 72.47 | 67.46 | 0.37 (-5.25, 5.98) | -4.41 (-10.33, 1.51) |  |  |  |  |
| CSE: upsetting * | IG | 58.93 | 67.04 | 74.86 | 9.57 (3.24, 15.91) ^✝^ | 15.98 (9.91, 22.05)^✝^ | 8.28 (-0.21, 16.76) | 0.056 | 18.49 (10.01, 26.98) | **<0.001** |
|  | UCG | 59.13 | 60.84 | 56.03 | 1.30 (-6.04, 8.63) | -2.52 (-9.98, 4.84 |  |  |  |  |
| Social support * | IG | 2.50 | 2.57 | 2.69 | 0.06 (-0.15, 0.26) | 0.19 (-0.01, 0.39) | 0.06 (-0.16, 0.29) | 0.579 | 0.25 (0.03, 0.48) | **0.028** |
|  | UCG | 2.72 | 2.69 | 2.65 | -0.01 (-0.22, 0.21) | -0.06 (-0.28, 0.15) |  |  |  |  |
| Behaviour Frequency * | IG | 1.74 | 1.86 | 2.55 | 0.11 (-0.10, 0.31) | 0.77 (0.57, 0.96)^✝^ | -0.09 (-0.27, 0.99) | 0.356 | -0.12 (-0.31, 0.06) | 0.200 |
|  | UCG | 1.60 | 1.80 | 2.51 | 0.20 (0.01, 0.38) ^✝^ | 0.89 (0.73, 1.04)^✝^ |  |  |  |  |
| Reactions Frequency * | IG | 1.53 | 1.39 | 1.77 | -0.14 (-0.33, 0.05) | 0.20 (0.01, 0.39) | -0.25 (-0.47, -0.03) | **0.029** | -0.25 (-0.47, -0.30) | **0.026** |
|  | UCG | 1.41 | 1.54 | 1.88 | 0.11 (-0.10, 0.32) | 0.46 (0.28, 0.63)^✝^ |  |  |  |  |
| QoL of PWD * | IG | 28.89 | 28.52 | 28.65 | -0.15 (-1.87, 1.57) | -0.18 (-1.81, 1.46) | 0.71 (-0.95, 2.37) | 0.406 | -0.90 (-2.56, 0.77) | 0.291 |
|  | UCG | 29.79 | 28.99 | 30.37 | -0.86 (-2.58, 0.86) | 0.72 (-0.93, 2.36) |  |  |  |  |
| Hospital admission %** | IG | 83.3 | 29.2 | 38.9 | 0.81 (0.45, 1.45) | 0.70 (0.38, 1.30) | 0.73 (0.31, 1.75) | 0.486 | 0.40 (0.16, 0.98) | **0.045** |
|  | UCG | 54.5 | 29.9 | 33.8 | 1.10 (0.58, 2.10) | 1.76 (0.91, 3.39) |  |  |  |  |
| Emergence department use %** | IG | 19.4 | 80.6 | 34.7 | 7.23 (3.68, 14.19) ^✝^ | 2.58 (1.18, 5.62) ^✝^ | 1.10 (0.40, 3.04) | 0.849 | 0.48 (0.16, 1.43) | 0.187 |
|  | UCG | 13.0 | 61.0 | 53.2 | 6.55 (3.07, 13.97) ^✝^ | 5.41 (2.49, 11.75) ^✝^ |  |  |  |  |

Note: (1) * a multivariate mixed effect linear regression model to estimate the intervention effects; (2) ** a mixed-effect Poisson regression model to determine the incidence rate ratio (IRR) between the two groups; (3) Hospital admission rate and Emergence department presentation rate were estimated based on one month data at baseline using Resource Utilization in Dementia (RUD) Questionnaire; (4) Abbreviations: UCG= usual care group; IG = iSupport group; M=months; MCS = mental component score; PCS = physical component score; QoL=Quality of Life; CSE= Caregiver self-efficacy; PWD=people with dementia. (5) Adjusted for confounders including gender, age, relationship with the care recipients, marital status, duration in care role, live in the same household with PWD or not, religion, employment status, education level, financial pressure, family members’ help and baseline outcome variables. (6) ✝The mean difference is statistically significant in the mixed-effect linear regression model in within-group comparisons.
