# Supplementary material for: Effects of a virtual iSupport Program on carers and people with dementia
Source: Alzheimers Dement. 2025 Sep 29;21(10):e70747. doi: 10.1002/alz.70747 (PMC12479211; doi:10.1002/alz.70747)
Supplement: Supplementary file 8 — Supporting Information [file ALZ-21-e70747-s007.docx]

**Supplementary file 7 Table S2: iSupport unit completion and satisfaction with the support**

| **Outcome measures** | **6 months** | **12 months** | **12 vs. 6 months differences (95% CI)** | **P** | **Total units: mean (SD, range)** | **Compliance with 20 units: %** |
| --- | --- | --- | --- | --- | --- | --- |
| iSupport unit completion: mean SD (range 0-30) | 7.18 (3.9) | 7.66 (3.83) | 0.11 (-0.06, 0.27) | 0.190 | 14.8 (5.27, 7-30) | 15.3 |
| Satisfaction with the intervention: mean SD (range 1-5) | 4.75 (0.59) | 4.85 (0.50) | 0.49 (-0.68, 1.66) | 0.412 | NA | NA |
